# Orai3 Regulates Pancreatic Cancer Metastasis by Encoding a Functional Store Operated Calcium Entry Channel

**DOI:** 10.3390/cancers13235937

**Published:** 2021-11-25

**Authors:** Samriddhi Arora, Jyoti Tanwar, Nutan Sharma, Suman Saurav, Rajender K. Motiani

**Affiliations:** 1 Laboratory of Calciomics and Systemic Pathophysiology (LCSP), Regional Centre for Biotechnology (RCB), Faridabad 121001, India; samriddhi.arora@rcb.res.in (S.A.); nutan.sharma@rcb.res.in (N.S.); suman.saurav@rcb.res.in (S.S.); 2 CSIR-Institute of Genomics and Integrative Biology (IGIB), New Delhi 110025, India; jyoti.tanwar@igib.in; 3 Academy of Scientific and Innovative Research (AcSIR), Ghaziabad 201002, India

**Keywords:** Orai3, store operated calcium entry, pancreatic cancer, metastasis

## Abstract

**Simple Summary:**

Pancreatic cancer (PC) is one of the most lethal forms of cancers with 5-year mean survival rate of less than 10%. Most of the PC associated deaths are due to metastasis to secondary sites. Calcium (Ca^2+^) signaling plays a critical role in regulating hallmarks of cancer progression including cell proliferation, migration and apoptotic resistance. Here, we demonstrate that a highly Ca^2+^ selective plasma membrane channel Orai3 is overexpressed in PC and is associated with poor prognosis in PC patients. Our data demonstrate that Orai3 modulates PC cell proliferation, apoptosis and migration. We further reveal that Orai3 regulates PC metastasis in immune-compromised mice. Collectively, our study establishes Orai3 as an attractive therapeutic target for managing PC metastasis, which may lead to better prognosis.

**Abstract:**

Store operated Ca^2+^ entry (SOCE) mediated by Orai1/2/3 channels is a highly regulated and ubiquitous Ca^2+^ influx pathway. Although the role of Orai1 channels is well studied, the significance of Orai2/3 channels is still emerging in nature. In this study, we performed extensive bioinformatic analysis of publicly available datasets and observed that Orai3 expression is inversely associated with the mean survival time of PC patients. Orai3 expression analysis in a battery of PC cell lines corroborated its differential expression profile. We then carried out thorough Ca^2+^ imaging experiments in six PC cell lines and found that Orai3 forms a functional SOCE channel in PC cells. Our in vitro functional assays show that Orai3 regulates PC cell cycle progression, apoptosis and migration. Most importantly, our in vivo xenograft studies demonstrate a critical role of Orai3 in PC tumor growth and secondary metastasis. Mechanistically, Orai3 controls G_1_ phase progression, matrix metalloproteinase expression and epithelial-mesenchymal transition in PC cells. Taken together, this study for the first-time reports that Orai3 drives aggressive phenotypes of PC cells, i.e., migration in vitro and metastasis in vivo. Considering that Orai3 overexpression leads to poor prognosis in PC patients, it appears to be a highly attractive therapeutic target.

## 1. Introduction

Pancreatic cancer (PC) is one of the deadliest cancers causing ~5 lakh deaths annually [1]. The 5-year mean survival rate of PC patients is less than 10% [2]. Further, PC incidence and mortality rate is increasing across the globe [2]. Most of the PC associated deaths are due to metastasis to the secondary sites, primarily the liver [3]. Therefore, it is critical to understand the molecular mechanisms that drive PC metastasis so that effective therapeutic strategies can be developed.

Calcium (Ca^2+^) signaling plays a critical role in tumorigenesis by regulating the hallmarks of cancer progression, such as cellular proliferation, invasion and metastasis [4,5]. Cancer progression is often associated with dysregulated Ca^2+^ homeostasis and altered functioning of Ca^2+^ handling proteins [4,5,6]. However, the role of Ca^2+^ dynamics in driving PC remains poorly appreciated. One of the most ubiquitous and highly regulated Ca^2+^ influx pathways is the store operated Ca^2+^ entry (SOCE) pathway. In non-excitable cells including pancreatic cells, SOCE mediated by Orai channels is the most important Ca^2+^ influx pathway [7,8,9,10]. SOCE is activated upon depletion of endoplasmic reticulum (ER) Ca^2+^ stores, which is sensed by ER Ca^2+^ sensors STIM1/STIM2 molecules via their EF-hand domain. In response to Ca^2+^ store depletion, STIM1/STIM2 molecules oligomerize and physically interact with plasma membrane Orai channels at ER-PM junctions. This communication leads to opening of the Orai channels and Ca^2+^ influx into the cells [11,12]. The Ca^2+^ entry by Orai channels drives vital cellular functions in a variety of cell types [13,14,15,16]. Both hypo and hyper functioning of Orai channels leads to pathological consequences [16,17].

In mammals, there are three Orai homologs (Orai1, 2 and 3) wherein Orai1 is the most studied among the three and plays a critical role in cellular physiology [16,18], while the functional significance of Orai2 and Orai3 is still emerging in nature [19,20,21]. The role of Orai1 in driving breast, lung, brain and prostate tumorigenesis is well documented [6,22]. Orai3, on the other hand, regulates the progression of certain specific subtypes of breast cancers by forming a functional SOCE channel [23,24,25,26]. Moreover, Orai3 can constitute store-independent channels by hetero-multimerizing with Orai1 [27,28,29,30]. Such channels can be activated by either arachidonic acid or its downstream metabolite leukotriene C_4_ and these channels are termed as ARC (arachidonic acid regulated Ca^2+^) channels or LRC (leukotriene C_4_ regulated Ca^2+^) channels, respectively [29,31]. The ARC channels were recently shown to mediate prostate cancer and PC progression [32,33]. However, the functional significance of Orai3 in PC remains poorly appreciated. Importantly, its role in driving PC metastasis is completely unexplored yet.

Here, we report that Orai3 expression is inversely associated with the mean survival time of PC patients wherein higher Orai3 expression leads to lower survival time. Our in vitro analysis suggests that Orai3 is differentially expressed in a battery of established PC cell lines. Interestingly, our data demonstrates that Orai3 forms a functional SOCE channel in PC cell lines. Furthermore, Orai3 regulates hallmarks of tumorigenesis, i.e., cell cycle progression, apoptosis and migration in vitro. Most importantly, our in vivo xenograft studies in immune-compromised mice clearly demonstrate a critical role of Orai3 in PC progression and metastasis. Mechanistically, Orai3 regulates G_1_-S phase transition in the cell cycle by controlling the expression of cyclin D1 and cyclin dependent kinase 4 (Cdk4). Further, Orai3 contributes to epithelial-mesenchymal transition (EMT) as evident with changes in the E-cadherin levels upon Orai3 knockdown. Moreover, Orai3 regulates expression of key metastasis associated matrix metalloproteinase (MMP) enzyme i.e. MMP2. Taken together, our study identifies Orai3 as a key regulator of PC metastasis, which is associated with poor prognosis in case of PC patients.

## 2. Materials and Methods

### 2.1. Materials and Reagents

Thapsigargin, 2APB and other cell culture reagents were purchased from Sigma. TRITC labeled annexin V was from AAT Bioquest. Fura-2AM was obtained from Invitrogen. The antibodies used were procured from Abcam, Cell Signaling Technology, Invitrogen and Santa Cruz Biotechnology. Please refer to Table 1 for the details of antibodies used in the study. shNT and shOrai3 were generously gifted by Prof. Mohamed Trebak (University of Pittsburgh, Pittsburgh, PA, USA). As reported earlier, these shRNAs were procured commercially and were cloned in the lentiviral vector pGIPZ [24].

List of Antibodies:

### 2.2. Cell Culture

Human pancreatic cancer cell lines BxPC3, Capan1, Capan2, CFPAC1, MiaPaCa2, Panc1 and primary human pancreatic cells (HPNE) were procured from ATCC. Cell lines BxPC3, Capan2 and CFPAC1 were cultured in RPMI-1640 with L-Glutamine (Lonza, Basel, Switzerland). MiaPaCa2 were cultured in DMEM medium supplemented with 10% fetal bovine serum (FBS) and 2.5% Horse serum. Panc1 were cultured in DMEM with 10% FBS. Primary pancreatic cell line hTERT HPNE were cultured in ATCC recommended media. Capan1 were maintained in Iscove’s modified dulbecco’s medium (IMDM) + 20% FBS.

### 2.3. Lentiviral Stable Cell Line Generation

For stable knockdown generation, human specific shOrai3 and shNT were cloned in the lentiviral pGIPZ vector (Dharmacon, Lafayette, CO, USA) were used. As reported earlier, the lentiviral constructs pCMV-VSVG, pCMV- dR8.2 and pGIPZ-shNT/shOrai3 were co-transfected in a flask containing 95% confluent HEK293FT cells [34]. Lipofectamine 2000 (Thermo Fisher, Waltham, MA, USA) was used as a transfection reagent to transfect HEK293FT cells. Viral particles containing medium were collected at 48 and 72 h after transfection and was concentrated using Amicon filters through centrifugation. These concentrated viral particles were used to transduce cells seeded at 50% confluency and knockdown was confirmed by performing Western blot analysis.

### 2.4. MTT Assay

Panc1 and HPNE lentiviral stables carrying either shNT or shOrai3 were seeded in a 96 well plate. After adherence, MTT reagent was added to cells at 4 different time points (24, 48, 72 and 96 h) and incubated at 37 °C for 3 h. After incubation for 3 h, media was aspirated and 100 µL of DMSO was added to dissolve purple colored formazan crystals. The absorbance was measured at 570 nm with a microplate reader.

### 2.5. Cell Cycle Analysis and Apoptosis Assay

Cell cycle analysis by quantitation of DNA is a standard application of flow cytometry. Panc1 lentiviral stables were seeded at a density of 80,000 cells/well in a 24 well plate. On the next day, the cells were incubated in serum free media for 12 h. The media was replaced by DMEM after 12 h. Cells were trypsinized and washed with PBS. For cells fixation, 70% ethanol was added for overnight and next day cells are washed with PBS carefully. Cells were incubated with RNase A at 37 °C for 40 min. Propidium iodide (PI) was added after RNase A treatment and FACS was performed. For the apoptotic assay, cells were stained with annexin V and PI and incubated for 30–60 min.

### 2.6. Calcium Imaging

Calcium imaging was performed as reported earlier [34,35]. Briefly, cells were cultured on confocal dishes for performing Ca^2+^ imaging. Cells are incubated at 37 °C for 30 min in a culture medium containing 4 μM fura-2 AM. After incubation, cells were washed 3 times and bathed in HEPES-buffered saline solution (140 mM NaCl, 1.13 mM MgCl_2_, 4.7 mM KCl, 2 mM CaCl_2_, 10 mM d-glucose, and 10 mM HEPES; pH 7.4) for ≥5 min before Ca^2+^ measurements were made. A digital fluorescence imaging system (Nikon Eclipse Ti2 microscope coupled with CoolLED pE-340 Fura light source and a high speed PCO camera) was used, and fluorescence images of several cells were recorded and analyzed. Fura-2AM was excited alternately at 340 and 380 nm, and the emission signal was captured at 510 nm. Figures showing Ca^2+^ traces are an average from several cells (the number of cells is denoted as “*n*” on each trace) attached on a single imaging dish. Each experiment was performed at least 3–4 times and the final data are plotted in the form of bar graphs. In the case of Panc1 stables, 9–11 runs on 3 independent days were performed. The data shown in a particular trace originates from multiple cells on a single imaging dish. The exact number of cells and traces for each condition are specified in the respective figure.

### 2.7. Western Blotting

Total protein was extracted with NP40 lysis buffer along with protease inhibitor cocktail. Total protein concentration was quantified by BCA assay. Protein extracts were separated by 8–12% SDS-PAGE and transferred to PVDF membranes. The membranes were blocked with 5% skimmed milk in Tris-buffered saline with Tween 20 (TBST) for 2 h at room temperature, and then incubated overnight at 4 °C in TBST with primary antibody, including rabbit anti-Orai3 (1:500, Abcam), rabbit anti-cyclin D1 (1:1000, Cell Signaling) and rabbit anti-Cdk4 (1:1000, Santa Cruz). Following incubation with horseradish peroxidase-conjugated donkey anti-rabbit secondary antibody (1:5000, NA934V, Sigma, St. Louis, MO, USA) for 2 h, the membranes were detected using the enhanced chemiluminescence (ECL) kit. β-tubulin (1:5000, Abcam) was used as a loading control. Densitometric analysis was done using ImageJ software and data are graphically represented as mean ± SEM.

### 2.8. Wound Healing Assay

Panc1 lentiviral stable cells were seeded in a 24 well plate at a density of 2 × 10^5^ cells per well. After 48 h of incubation, cells reached 100% confluency and a wound was created at the center of the well by gently scrapping the cells with a sterile P200 tip. After removing the incubation media and washing with PBS, new media were added to each well. Each well was observed under the bright field microscope for migration at different time points. The migration rate of individual conditions (shNT and shOrai3) was determined by measuring the distance covered from the initial timepoint to selected timepoints. Different widths were normalized to the 0 h timepoint. Graph was plotted for percentage wound healing vs. time (h) using GraphPad Prism software. Data are presented as mean ± SEM.

### 2.9. Migration Assay

Cell migration was assessed using 24-well corning costar inserts with 8-μm pores. Cells (5 × 10^4^) were added in the upper chamber in DMEM media (without FBS) and incubated at 37 °C and migration was assessed at 24 h. Non-migrating cells were removed from the upper chamber with the help of cotton buds. Cells adhered at the bottom of the transwell were fixed with formaldehyde, permeabilized with 100% methanol and stained with crystal violet and bright field images of 5 different fields were quantified using ImageJ. Experiments were performed in triplicate in three independent biological samples and data are reported as mean ± SEM.

### 2.10. In Vivo Experiments

Animal protocols were approved by the Institutional Animal Ethics Committee (IAEC), Regional Center for Biotechnology (Protocol #RCB/IAEC/2020/076). Four-to-six weeks old female NOD SCID mice were acclimated at the Small Animal Facility (SAF) for 2 weeks before any intervention. 1 × 10^6^ Panc1 stables either shNT or shOrai3 in a volume of 100 µL with 100 µL of Matrigel were injected subcutaneously into the left flank of 5 mice/condition. Tumor volume was assessed weekly using Vernier calipers. Tumor volume was calculated using the formula *V = W*^2^ × *L*/2, where *V* is tumor volume, *W* is tumor width, and *L* is tumor length [24]. Animals were euthanized 12 weeks post injection and pictures of isolated tumors were taken. Tumor weights were measured using a digital scale.

For metastasis studies, we performed whole body fluorescence imaging using the SPECTRUM In Vivo Imaging System (IVIS) (PerkinElmer, Santa Clara, CA, USA) by setting the excitation filter at 500 nm and the emission filter at 540 nm. Animals were anesthetized using 1.5% isoflurane in the anesthesia chamber and were transferred to IVIS for imaging. Whole body and thoracoabdominal region fluorescence imaging was performed for evaluating the tumor development and metastasis, respectively.

### 2.11. Tumor Lysates Preparation

Tumors were harvested from two groups (shNT and shOrai3) NOD SCID mice and were snap frozen using liquid nitrogen. Subsequently, they were thawed on ice. A portion of the tumor was cut with the help of a sterile scalpel and weighed before adding an appropriate amount of protein lysis buffer (NP40 + protease inhibitor cocktail). Tumor samples suspended in lysis buffer were homogenized using hand-held tissue homogenizer on ice and further centrifuged at 13,000 rpm at 4 °C to obtain protein lysate. The supernatant obtained after centrifugation was used to estimate the total protein concentration by performing BCA assay and protein lysates were used for performing Western blotting.

### 2.12. qRT-PCR Analysis

For mRNA extraction Panc1 cells stably expressing either shNT or shOrai3 were processed with Qiagen RNeasy kit (Catalog #74106). mRNA was then converted to cDNA using high-capacity cDNA reverse transcription kit from ThermoFisher (Waltham, MA, USA) (Catalog #4368814). Real-time PCR reactions were performed using SYBR green in Quant Studio 6 Flex from Applied Biosystems. The data were analyzed with Quant Studio real-time PCR software version 1.3. The expression of Orai1, Orai2 and Orai3 in shNT and shOrai3 cells were normalized to that of the housekeeping gene GAPDH. The sequences of primers used in the study are listed in the Table 2.

### 2.13. Statistical Analysis

All the experiments were performed at least three times. Data are presented as mean ± SEM and unpaired Student’s *t*-test was performed for determining the statistical significance. For Figure 6, a paired *t*-test was performed while for in vivo tumor volume data (presented in the Figure 8) a two-way ANOVA was performed. *p*-value < 0.05 was considered as significant and is presented as “*”; *p*-value < 0.01 is presented as “**”and *p*-value < 0.001 is presented as “***”.

## 3. Results

### 3.1. Orai3 Expression Is Inversely Correlated to the Pancreatic Cancer Patients Survival

**Figure 1:**
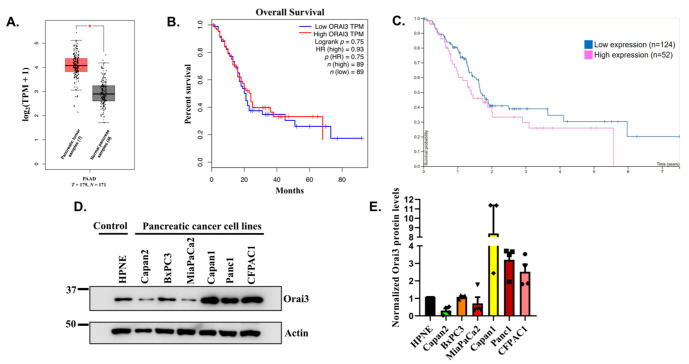
Orai3 expression is inversely associated with the mean survival time of pancreatic cancer patients. (**A**) Orai3 expression analysis in GEPIA (Gene Expression Profiling Interactive Analysis) database having 179 pancreatic tumor samples (*T*) and 171 normal pancreas samples (*N*). Here “*” indicates that Orai3 expression is significantly higher in pancreatic tumor samples in comparison to normal pancreas samples. (**B**) Pancreatic cancer patients’ survival analysis in GEPIA wherein blue trace corresponds to low Orai3 expression (*n* = 89 patients) and red trace represents high Orai3 expression (*n* = 89 patients) clearly suggesting that higher Orai3 expression is associated with less patient survival time. (**C**) Pancreatic cancer patients’ survival analysis in “The Human Protein Atlas” database where blue trace corresponds to low Orai3 expression (*n* = 124 patients) and red trace represents high Orai3 expression (*n* = 52 patients) demonstrates that higher Orai3 levels are associated with poor prognosis and leads to decrease in patients’ survival time. (**D**) Western blot analysis for examining Orai3 protein levels in well-established pancreatic cancer cell lines and transformed “normal” pancreatic cells. (**E**) ImageJ based densitometric analysis of Orai3 expression in pancreatic cancer cell lines suggest differential Orai3 expression profile (*n* = 4).

For delineating the role of Orai3 in PC progression, we performed expression analysis of this Ca^2+^ channel using publicly available databases “GEPIA” (Gene Expression Profiling Interactive Analysis) and “The Human Protein Atlas”. GEPIA is an interactive web server for investigating RNA sequencing expression of 9736 tumors and 8587 normal samples from the TCGA (The Cancer Genome Atlas) and the GTEx (genotype-tissue expression) projects. Our extensive bioinformatics analysis found that Orai3 is significantly overexpressed in PC tissue samples (*n* = 179 patients) in comparison to healthy pancreases (*n* = 171 healthy individuals) (Figure 1A). Next, we evaluated the association of higher Orai3 expression to the mean survival time of the PC patients using “GEPIA” and “The Human Protein Atlas”. Interestingly, analysis of both the datasets suggested that higher Orai3 expression is associated with poor prognosis and less survival time (Figure 1B,C). The “GEPIA” analysis for 89 samples with high Orai3 expression and 89 samples with relatively low Orai3 levels demonstrated that none of the PC patients with higher Orai3 expression could survive beyond 5.5 years while, ~20% PC patients with low Orai3 expression survived more than 7.5 years (Figure 1B). We further performed similar analysis of “The Human Protein Atlas” dataset with 52 PC samples with relatively higher Orai3 protein expression and 124 PC samples showing low Orai3 levels. We observed a strikingly analogous trend in “The Human Protein Atlas” dataset wherein all the PC patients with higher Orai3 protein expression in the PC tumors succumbed by 5.5 years whereas more than 20% of patients with low Orai3 levels in the PC tumors survived for more than 7.5 years (Figure 1C). This clinically relevant observation of inverse association of Orai3 expression with PC patient survival time, which emanated from two independent and unbiased datasets clearly suggests that Orai3 plays a critical role in PC progression, metastasis and associated mortality. Therefore, we started elucidating the precise role of Orai3 in PC progression with special emphasis on PC metastasis.

### 3.2. Orai3 Is Expressed in “Normal” as Well as Cancerous Pancreatic Cell Lines and Forms a Functional Ca^2+^ Influx Channel

First of all, we examined the Orai3 protein expression in a battery of PC cell lines (BxPC3, Capan1, Capan2, CFPAC1, MiaPaCa2 and Panc1) and compared it with the Orai3 levels in immortalized “normal human pancreatic epithelial cells-HPNE”. All these cell lines were procured from “American Type Culture Collection” (ATCC). We performed Western blot analysis on the protein lysates of “normal” pancreatic epithelial cells and 6 PC cell lines for evaluating Orai3 expression (Figure 1D). Similar to publicly available datasets, we observed a differential expression of Orai3 in these cell lines. In comparison to HPNE cells, it was found that: (1) Orai3 levels were higher in Capan1, CFPAC1, and Panc1; (2) Capan2 and MiaPaCa2 showed lower Orai3 expression; and (3) while in BxPC3, Orai3 levels were comparable to that in HPNE cells (Figure 1D,E). This expression analysis corroborates the unbiased largescale publicly available datasets from different sources, i.e., GEPIA, TCGA and The Human Protein Atlas. Taken together, it suggests that Orai3 is overexpressed in a certain proportion of PC tumors and it is not universally upregulated in all PC samples.

**Figure 2:**
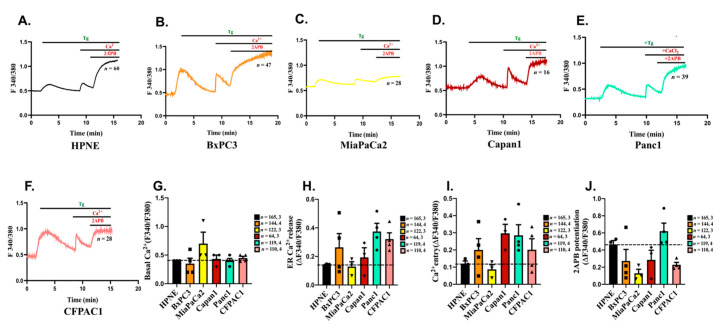
Characterization of SOCE in a battery of Pancreatic cancer cell lines. (**A**–**F**) Representative Ca^2+^ imaging traces of HPNE, BxPC3, MiaPaCa2, Capan1, Panc1 and CFPAC1 cells where “n” denotes the number of cells in that particular trace. (**G**) Quantitation of basal Ca^2+^ levels across different cell lines. (**H**) The amplitude of ER Ca^2+^ release was calculated from a number of experiments and data are presented in dot plot graphs. (**I**) The extent of SOCE was calculated from several experiments and data are presented in dot plot graphs. (**J**) 2-APB induced potentiation of Orai3 mediated SOCE was calculated from several experiments and data are presented in dot plot graphs. The total number of cells imaged in (**G**–**J**) are reported as “*n*  =  *x*, *y*” where “*x*” denotes total number of cells imaged and “*y*” denotes number of traces recorded.

**Figure 3:**
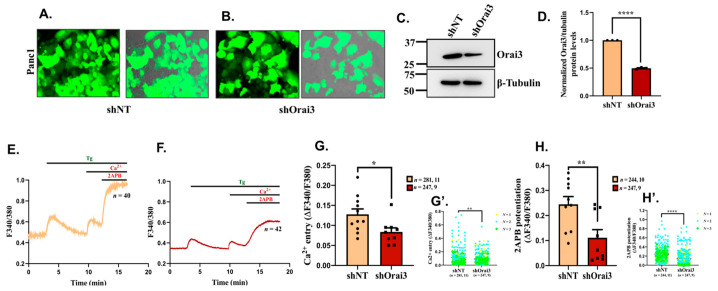
Orai3 encodes a functional SOCE channel in Panc1 cells. (**A**,**B**) Lentiviral transduced Panc1 cells stably expressing either GFP tagged shNT control or GFP tagged shRNA targeting Orai3 (fluorescence and merged images of bright field plus fluorescence are presented). (**C**) Representative Western blot showing knockdown of Orai3 in shOrai3 Panc1 stables in comparison to shNT control Panc1 stable cells. (**D**) Densitometric analysis of Orai3 silencing in 3 independent shNT and shOrai3 Panc1 stable clones. (**E**) Representative Ca^2+^ imaging trace of shNT Panc1 stables where “*n* = 40” denotes the number of cells in that particular trace. (**F**) Representative Ca^2+^ imaging trace of shOrai3 Panc1 stables where “*n* = 42” denotes the number of cells imaged in that particular trace. (**G**) The extent of SOCE was calculated from 281 shNT and 247 shOrai3 cells, which were imaged from several autonomous experiments/traces (11 traces of shNT and 9 traces of shOrai3 originating from 3 independent clones of shNT and shOrai3 Panc1 stables) and data from autonomous traces are presented in dot plot graphs. (**G’**) Data for extent of SOCE from all individual cells from 3 independent clones are presented as clone N°1 yellow rounds, clone N°2 blue squares, clone N°3 green diamond. (**H**) 2-APB induced potentiation of Orai3 mediated SOCE was calculated from around 250 cells/condition. These cells were imaged during several autonomous experiments/traces (10 traces of shNT and 9 traces of shOrai3 originating from 3 independent clones of shNT and shOrai3 Panc1 stables) and data are presented in dot plot graphs. (**H’**) Data for 2-APB induced SOCE potentiation from all individual cells from 3 independent clones are presented as clone N°1 yellow rounds, clone N°2 blue squares, clone N°3 green diamond. The total number of cells imaged are reported in (**G**,**H**) as “*n*  =  x, y” where “x” denotes total number of cells imaged and “y” denotes number of traces recorded. Data presented are mean ± S.E.M. Unpaired Student’s *t*-test was performed for statistical analysis. *p*-value < 0.05 was considered as significant and is presented as “*”; *p*-value < 0.01 is presented as “**”and *p*-value < 0.0001 is presented as “****”.

**Figure 4:**
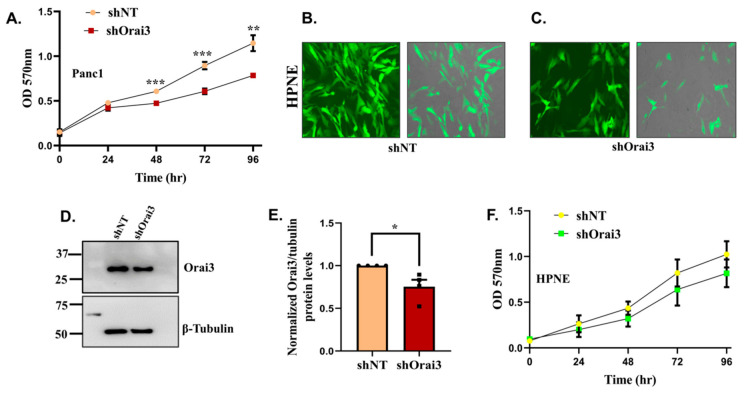
Orai3 regulates Panc1 but not HPNE cell viability. (**A**) MTT assay-based cell viability analysis (24–96 h time points) in Panc1 shNT and Panc1 shOrai3 stable cells (*n* = 5). (**B**,**C**) Lentiviral transduced HPNE cells stably expressing either GFP tagged shNT control or GFP tagged shRNA targeting Orai3 (fluorescence and merged images of bright field plus fluorescence are shown). (**D**) Representative Western blot showing knockdown of Orai3 in shOrai3 HPNE stables in comparison to shNT control HPNE stable cells. (**E**) Densitometric analysis of Orai3 silencing in 4 independent shNT and shOrai3 HPNE stable clones. (**F**) MTT assay-based cell viability analysis (24–96 h time points) in HPNE shNT and HPNE shOrai3 stable cells (*n* = 4). Data presented as mean  ±  S.E.M. Unpaired Student’s *t*-test was performed for statistical analysis. *p*-value < 0.05 was considered as significant and is presented as “*”; *p*-value < 0.01 is presented as “**”and *p*-value < 0.001 is presented as “***”.

We next evaluated the functional significance of Orai3 expression in “normal” pancreatic epithelial cells and PC cell lines by performing live cell Ca^2+^ imaging with ratio-metric Fura2-AM dye. We employed standard thapsigargin (Tg) activated SOCE protocol [34,35] wherein ER Ca^2+^ stores were depleted by Tg in absence of extracellular Ca^2+^ and subsequent addition of Ca^2+^ in the bath solution resulted in SOCE. Further, we utilized the pharmacological tool 2-Aminoethoxydiphenyl borate (2APB) to differentiate between functional Orai1 v/s Orai3 channels. It is well reported that 2APB in the same concentration range of 30–50 µM blocks Orai1 and activates Orai3 channels [36,37,38,39]. In the past, several groups used 2APB (50 µM) to demonstrate Orai3 activation as a functional SOCE channel in breast and lung cancers [20,23,24,40,41]. Therefore, in order to examine the functionality of Orai3 as a SOCE channel in pancreatic cells, we used 2APB (50 µM) in our live cell Ca^2+^ imaging experiments. Interestingly, our Ca^2+^ imaging studies in the “normal” HPNE and PC cell lines show that the SOCE is potentiated by 2APB in these cells suggesting that Orai3 is most likely the functional SOCE channel in these cells (Figure 2A–F). We performed these experiments multiple times with >100 cells and further analyzed the Ca^2+^ imaging data in detail. The Ca^2+^ imaging data was analyzed as per the standards of the field, which are followed by several leading groups across the globe. Briefly, the basal cytosolic levels were calculated at the start of the experiment and plotted as ratio of fluorescence emission intensity acquired upon excitation of Fura-2 at 340 nm and 380 nm. The ER Ca^2+^ release was measured by calculating the rise in the ratio of fluorescence emission intensity upon addition of Tg. We then waited until the cytosolic Ca^2+^ levels reached to the baseline and subsequently added Ca^2+^ in the bath solution. SOCE was quantitated by determining the increase in the ratio of fluorescence emission intensity upon the addition of Ca^2+^ in the bath solution. Upon the plateauing of SOCE, we added 2APB (50 µM) for examining the consequence of 2ABP addition on the SOCE. Finally, 2APB potentiation was measured by analyzing the rise in the ratio of fluorescence emission intensity upon 2APB addition. Our analysis shows that: (1) basal cytosolic Ca^2+^ levels were comparable across these cells (Figure 2G); (2) ER Ca^2+^ release (the indirect measure of ER Ca^2+^ stores) was higher in PC cells, except MiaPaCa2, as compared to HPNE cells (Figure 2H); (3) similarly, in comparison to HPNE cells, SOCE was greater in PC cells except MiaPaCa2 (Figure 2I); (4) finally, SOCE was potentiated by 2APB in all these cell lines wherein highest potentiation was observed in case of Panc1 and lowest in case of MiaPaCa2 (Figure 2J). It is important to note that the Panc1 cells express higher Orai3 expression in comparison to HPNE whereas MiaPaCa2 cells express lower Orai3 levels. Since Orai3 is overexpressed and most likely forms a functional channel in Panc1 cells, we performed further validation studies in Panc1 cells.

For corroborating the role of Orai3 in SOCE, we generated lentiviral-based stable Panc1 cell lines expressing either non-targeting control shRNA (shNT) or shRNA specifically targeting Orai3 (shOrai3). We used the shRNA vectors co-expressing GFP for fluorescence-based identification of transduced cells and for performing in vivo bio-fluorescence studies (Figure 3A,B). First of all, we confirmed Orai3 silencing in shOrai3 stable Panc1 cell line by performing Western blot analysis (Figure 3C). We observed a significant knockdown of Orai3 in shOrai3 stable cells in comparison to shNT cells (Figure 3D). Next, we examined the specificity of shOrai3 by analyzing the mRNA and protein expression of Orai3 homologs, i.e., Orai1 and Orai2 in shOrai3 Panc1 stable cells. As presented in Supplementary Figure S1A, in Panc1 shOrai3 stables Orai3 mRNA levels were decreased by over 50% in comparison to that in shNT Panc1 cells. Whereas Orai1 and Orai2 mRNA levels remain comparable between shNT and shOrai3 Panc1 cells (Figure S1A). We also corroborated shOrai3 specificity at protein levels by performing Western blot analysis for Orai1 and Orai2 in Panc1 stable cells. As expected, we observed similar levels of Orai1 and Orai2 in shNT versus shOrai3 Panc1 stable cells (Figure S1B–E). Taken together, these experiments clearly demonstrate that shOrai3 is specific to Orai3. After confirming specificity, we used shNT and shOrai3 Panc1 stable cells for performing live cell Ca^2+^ imaging experiments. We performed Ca^2+^ imaging experiments in around 250 cells/condition. As presented in Figure 3E, shNT Panc1 stable cells showed SOCE that was potentiated upon 2APB application whereas shOrai3 Panc1 stable cells displayed lower SOCE as well as reduced 2APB mediated potentiation (Figure 3F). The detailed analysis of Ca^2+^ imaging traces demonstrate that Orai3 knockdown in Panc1 cells significantly decrease both SOCE and 2APB induced SOCE potentiation (Figure 3G,H). Interestingly, 2APB (50 µM) can activate Orai3 independent of Ca^2+^ store depletion. Therefore, we performed additional Ca^2+^ imaging assays in shNT and shOrai3 Panc1 stable cells wherein we directly stimulated cells with 2APB (50 µM) without Tg pre-exposure. As shown in Supplementary Figure S2A,B, 2APB activated Ca^2+^ influx in shNT Panc1 stables and this influx was significantly decreased in shOrai3 Panc1 cells. We analyzed 2APB stimulated store independent Ca^2+^ entry in around 150 cells/condition. The quantitation of this data is presented in Supplementary Figure S2C. Collectively, our live cell Ca^2+^ imaging experiments clearly show that Orai3 is a functional SOCE channel in Panc1 cells.

### 3.3. Orai3 Regulates PC Cell Viability, Cell Cycle Progression and Apoptosis

For understanding the role of Orai3 in PC progression, we started by evaluating Orai3 contribution to PC cell viability. We performed a colorimetry-based MTT assay for examining the significance of Orai3 in Panc1 viability. As presented in Figure 4A, the cell viability was significantly decreased in shOrai3 Panc1 stables in comparison to shNT stables. We next sought to determine if Orai3′s role in regulating cell viability is specific to PC cells or is it conserved in “normal” pancreatic epithelium. Therefore, we generated lentiviral-based stable silencing of Orai3 in HPNE cells. We used similar strategy as discussed above for Panc1 cells and generated shNT control stable HPNE cells and shOrai3 stable HPNE cells (Figure 4B,C). We performed Western blot analysis to confirm the knockdown of Orai3 in HPNE cells (Figure 4D) and observed that there was a significant reduction in Orai3 expression in shOrai3 HPNE stable cells in comparison to shNT control cells (Figure 4E). We then performed MTT assays on these HPNE stable cells and found that Orai3 knockdown only marginally (non-significantly) affected the viability of “normal” pancreatic cells (Figure 4F). This suggests that Orai3 specifically contributes to the cellular viability of PC cells. We thus performed further functional assays with Panc1 shNT and shOrai3 stable cells.

**Figure 5:**
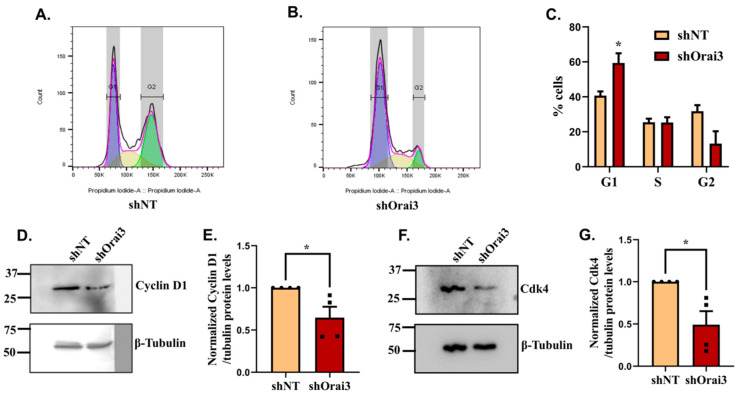
Orai3 contributes to Panc1 cell-cycle progression. (**A**,**B**) Representative data showing FACS-based cell-cycle analysis of Panc1 shNT and Panc1 shOrai3 stable cells. Cell-cycle analysis was performed with three independent biological experiments using propidium iodide. (**C**) Quantitative analysis of % cells in different phases of cell cycle in the case of Panc1 shNT and Panc1 shOrai3 stable cells (*n* = 4). (**D**) Western blot analysis for cyclin D1 expression in shNT and shOrai3 Panc1 stable cell lines. (**E**) Densitometric analysis of cyclin D1 levels in shNT and shOrai3 Panc1 stable cells. (**F**) Western blot analysis for Cdk4 expression in shNT and shOrai3 Panc1 stable cell lines. (**G**) Densitometric analysis of Cdk4 levels in shNT and shOrai3 Panc1 stable cells. Data presented as mean ± S.E.M. Unpaired Student’s *t*-test was performed for statistical analysis. *p*-value < 0.05 was considered as significant and is presented as “*”.

The decrease in cell viability observed upon Orai3 knockdown in Panc1 cells could be either due to decrease in cell cycle progression or increase in basal cell apoptosis. Thus, we performed both cycle cell analysis and cell apoptosis estimation in shNT and shOrai3 Panc1 cells. We carried out standard propidium iodide-based FACS measurements of the cell-cycle progression in Panc1 shNT and shOrai3 cells (Figure 5A,B). Interestingly, we observed that Orai3 silencing led to accumulation of Panc1 cells in the G_1_ phase of cell cycle and there were substantially less cells in G_2_ phase in comparison to shNT stable cells (Figure 5C). In shNT Panc1 stables, cells were able to transit to the S and G_2_ phases of the cell cycle while in the case of shOrai3 stables, the majority of cells (>50%) were stalled in the G_1_ phase suggesting that Orai3 silencing induces cell-cycle arrest in the G_1_ phase (Figure 5C). To further corroborate this observation, we examined the levels of key G_1_ phase transition regulators, i.e., cyclin D1 and cyclin dependent kinase 4 (Cdk4) in shOrai3 stables. We performed Western blot analysis in shNT and shOrai3 stables for evaluating cyclin D1 and Cdk4 expression. As expected, the expression of both cyclin D1 (Figure 5D,E) and Cdk4 (Figure 5F,G) was significantly decreased in shOrai3 stables in comparison to shNT control cells suggesting that Orai3 plays a critical role in the G_1_ phase transition of PC cells.

**Figure 6:**
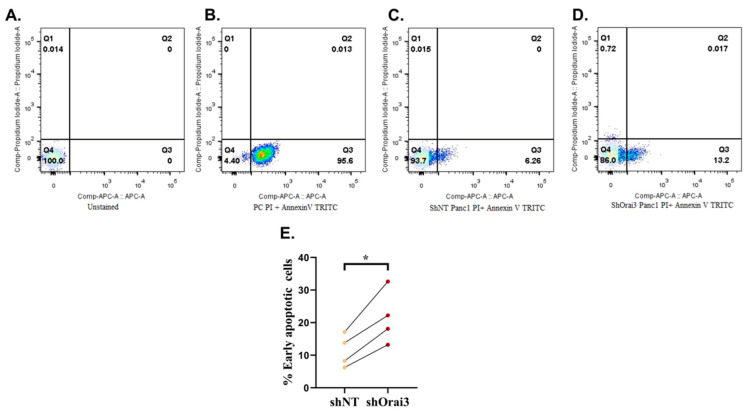
Orai3 regulates basal apoptosis of Panc1 cells. (**A**–**D**) Representative FACS-based analysis of apoptosis using propidium iodide and TRITC conjugated annexin V for unstained, i.e., negative control Panc1 cells (**A**); positive control Panc1 cells (**B**); shNT Panc1 stable cells (**C**) and shOrai3 Panc1 stable cells (**D**). (**E**) Quantitative analysis of % early apoptotic cells in shNT and shOrai3 Panc1 stable cell lines from 4 independent experiments. Data presented as mean ± S.E.M. Paired *t*-test was performed for statistical analysis. *p*-value < 0.05 was considered as significant and is presented as “*”.

Next, we examined the role of Orai3 in modulating PC cell apoptosis by performing standard annexin V-based FACS assays. Since our stable cells were constitutively expressing GFP, we used TRITC conjugated annexin V to ensure that there was no spectral overlap between live and apoptotic cells in our experiments. This strategy helped in ruling out the possibility of any false positive apoptotic signals during FACS analysis. We used unstained cells and cells heated at 95 °C as negative and positive controls, respectively, in the apoptosis assays. As presented in Figure 6A,B, we observed 0% and 95.6% early apoptotic cells in the negative and positive controls, respectively. It suggests that our assay was working efficiently, and we were able to capture correct biological signals using TRITC conjugated annexin V. We then evaluated basal cellular apoptosis in shNT (Figure 6C) and shOrai3 Panc1 stables (Figure 6D) and found that there was a two-fold increase in the number of apoptotic cells in shOrai3 condition as compared to shNT cells (Figure 6E). Taken together, this data suggests that Orai3 plays a vital role in PC cell survival and therefore, Orai3 silencing leads to significant increase in the cellular apoptosis of Panc1 cells.

### 3.4. Orai3 Controls PC Cell Migration In Vitro

**Figure 7:**
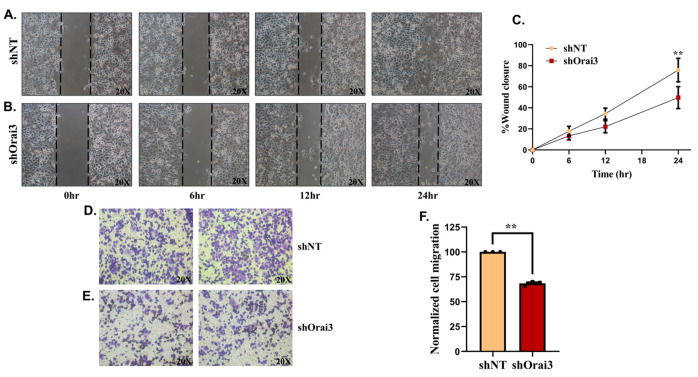
Orai3 controls migration of Panc1 cells. Scratch wound healing (**A**–**C**) and transwell migration assays (**D**–**F**) were performed for evaluating the role of Orai3 in regulating Panc1 migration. (**A**) Representative wound healing images at 0, 6, 12 and 24 h time points in the case of shNT Panc1 stable cells. (**B**) Representative wound healing images at 0, 6, 12 and 24 h time point in case of shOrai3 Panc1 stable cells. (**C**) Quantitative analysis of %wound healing over the period of 24 h in shNT and shOrai3 Panc1 stable cells from 4 independent experiments. (**D**) Representative micrographs of crystal violet stained transwell migrated shNT Panc1 stable cell line. (**E**) Representative micrographs of crystal violet stained transwell migrated shOrai3 Panc1 stable cells. (**F**) Quantitative analysis of number of migrated cells in the transwell migration assay from three independent experiments. Data presented as mean ± S.E.M. Unpaired Student’s *t*-test was performed for statistical analysis. *p*-value < 0.05 was considered as significant and *p*-value < 0.01 is denoted as “**”.

Since most of the PC associated deaths are due to PC metastasis to the secondary sites, we started evaluating the relevance of Orai3 in PC cell migration. We first utilized scratch wound assays for examining the role of Orai3 in Panc1 cell migration. We allowed shNT and shOrai3 stable Panc1 cells to form a monolayer in 24-well plates and then created a wound in the monolayer using 200 µL pipette tip. We observed the wound closure, which gave an estimation of cellular migration at regular intervals until the 24-h timepoint. It is important to mention that the doubling time of Panc1 cells is >50 h (documented at the ATCC website as well). Therefore, in this assay our observations were specific to Panc1 cell migration. As shown in Figure 7A,B, shNT Panc1 stable cells were able to migrate and close the wound more efficiently than shOrai3 Panc1 stables. We performed this assay in triplicates with four independent biological samples and observed that shNT Panc1 stables were able to close around 80% of the wound in 24 h whereas shOrai3 cells closed just over 40% of the wound in the same time (Figure 7C). This suggests that Orai3 could be playing an important role in driving PC-cell migration. To further substantiate this observation, we performed trans-well Boyden chamber-based migration studies with shNT and shOrai3 stable Panc1 cells. We seeded either shNT or shOrai3 cells in the top chamber (with 8 µm pores) containing culture media without serum and used complete media including serum in the bottom chamber. Therefore, in these assays, serum which is rich in growth factors served as a chemoattractant. We allowed cells to migrate towards the lower chamber for 24 h and fixed the migrated cells using formaldehyde. Eventually, migrated cells were stained with crystal violet and quantitation of number of migrated cells was performed using ImageJ software. As presented in Figure 7D,E, the cell migration (crystal violet staining) is substantially less in the case of shOrai3 stables in comparison to shNT stables. Further, the quantitation of data from three independent biological samples suggests that Orai3 silencing results in around 35% decrease in the Panc1 cells migration in trans-well assays (Figure 7F). Taken together, both wound healing and trans-well migration assays highlight a crucial role for Orai3 in regulating PC-cell migration.

### 3.5. Orai3 Regulates PC Progression and Metastasis In Vivo

**Figure 8:**
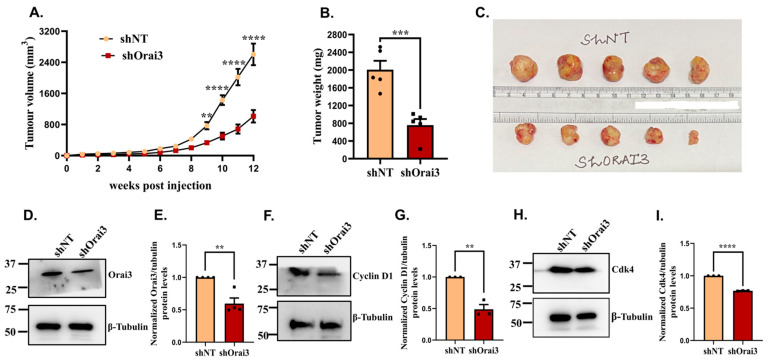
Orai3 regulates pancreatic cancer progression in vivo. (**A**) Weekly tumor volume measurements in NOD SCID mice injected with either shNT Panc1 cells or shOrai3 Panc1 cells (*n* = 5 mice/condition). (**B**). Tumor weight measurements after sacrificing mice at 12-week post-injections (either shNT Panc1 or shOrai3 Panc1 injections) timepoint. (**C**). Pictures of shNT and shOrai3 Panc1 tumors harvested after 12 weeks of injections. (**D**). Western blot analysis for Orai3 expression in shNT Panc1 and shOrai3 Panc1 tumors. (**E**). Densitometric analysis of validating Orai3 knockdown in shOrai3 Panc1 tumors in comparison to shNT Panc1 tumors. (**F**). Western blot analysis for cyclin D1 levels in shNT Panc1 and shOrai3 Panc1 tumors. (**G**). Densitometric analysis of cyclin D1 levels in shNT Panc1 and shOrai3 Panc1 tumors. (**H**). Western blot analysis for Cdk4 expression in shNT Panc1 and shOrai3 Panc1 tumors. (**I**) Densitometric analysis of Cdk4 levels in shNT Panc1 and shOrai3 Panc1 tumors. Data presented as mean ± S.E.M. Unpaired Student’s *t*-test was performed for statistical analysis except from tumor volume wherein two-way ANOVA was performed. *p*-value < 0.01 is denoted as “**”; *p*-value < 0.001 is presented as “***” and *p*-value < 0.0001 is denoted as “****”.

Finally, we performed in vivo studies in immuno-compromised NOD SCID mice for determining the role of Orai3 in PC progression and metastasis. We subcutaneously injected 1 × 10^6^ cells, either shNT or shOrai3 Panc1 stables, in 5 female NOD SCID mice/condition. We followed the tumor growth by measuring the tumor volumes in these mice on a weekly basis. In the beginning, tumor development was slow, and after 4 weeks, tumor growth commenced at a rapid rate (Figure 8A). We used digital Vernier calipers for measuring tumor sizes and followed tumor growth for 12 weeks. As presented in Figure 8A, the tumor volume in case of shNT Panc1 stables was drastically higher than that in the case of shOrai3 Panc1 stables. We sacrificed the mice at the 12-week timepoint and harvested tumors from them. We next performed tumor weight analysis and observed that there was around a 60% reduction in tumor weight in the case of shOrai3 Panc1 stables in comparison to shNT Panc1 stables (Figure 8B). The noteworthy reduction in tumor volume and weight was also evident from the pictures of harvested tumors (Figure 8C). This data clearly establishes Orai3 as a critical regulator of PC progression in vivo.

We then attempted to understand the molecular mechanism driving the decrease in the tumor growth in vivo. In our in vitro experiments, we observed that Orai3 regulates cyclinD1 and Cdk4 expression and thereby stall cell-cycle progression. Therefore, we examined if this signaling cascade is functional in the in vivo setting as well. We lysed the snap frozen tumors for making protein samples and performed Western blot analysis on the tumor lysates. First of all, we evaluated the expression of Orai3 in shNT and shOrai3 tumors. We detected close to a 45% reduction in the Orai3 levels in shOrai3 tumors in comparison to shNT tumors (Figure 8D,E). We next examined the levels of cyclinD1 and Cdk4 in shNT and shOrai3 tumor protein samples. In concordance with the in vitro data, the expression of both cyclinD1 (Figure 8F,G) and Cdk4 (Figure 8H,I) was significantly reduced in shOrai3 tumor protein samples in comparison to that in shNT tumor lysates. In summary, this suggests that Orai3 regulates PC tumor growth at least in part by controlling cell-cycle progression.

**Figure 9:**
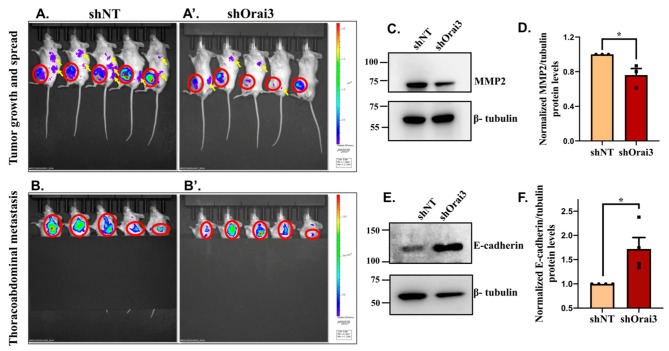
Orai3 regulates pancreatic cancer metastasis. (**A**) Whole-body bio-fluorescence imaging signals at 12-week post-injection timepoint in the case of shNT Panc1 stables injected mice. (**A’**) Whole-body bio-fluorescence imaging signals in shOrai3 Panc1 stable cells injected mice at 12-week post-injection timepoint. The initial xenografts at the site of injections are marked in red circles while metastatic spread is highlighted with yellow arrows. (**B**) Thoracoabdominal region bio-fluorescence imaging signals at 12-week post-injection time-point in case of shNT Panc1 stables injected mice. (**B’**) Thoracoabdominal region bio-fluorescence imaging signals in shOrai3 Panc1 stable cells injected mice at 12-week post-injection timepoint. The thoracoabdominal bio-fluorescence imaging signals are identified in red circles. The time interval between whole body and thoracoabdominal bio-fluorescence imaging was 3 to 5 min. (**C**) Western blot analysis for MMP2 protein expression in shNT Panc1 and shOrai3 Panc1 tumors. (**D**) Densitometric analysis of MMP2 levels in shNT Panc1 and shOrai3 Panc1 tumors. (**E**) Western blot analysis for E-cadherin protein expression in shNT Panc1 and shOrai3 Panc1 tumors. (**F**) Densitometric analysis of E-cadherin levels in shNT Panc1 and shOrai3 Panc1 tumors. Data presented as mean ± S.E.M. Un-paired *t*-test was performed for statistical analysis. *p*-value < 0.05 was considered as significant and is presented as “*”.

We next investigated the role of Orai3 in PC metastasis by performing live animal bio-fluorescence imaging. Since we injected either GFP expressing shNT or GFP expressing shOrai3 Panc1 stable cells in mice, we utilized the powerful live animal bio-fluorescence imaging for examining the GFP signals coming from mice. It is important to highlight that mice do not express GFP on their own and therefore bio-fluorescence of GFP during animal imaging directly corresponds to the presence of GFP positive Panc1 cells. We performed whole-body fluorescence imaging using the SPECTRUM In Vivo Imaging System (IVIS) (PerkinElmer, Santa Clara, CA, USA). Animals were anesthetized using 1.5% isoflurane in the anesthesia chamber and were transferred to IVIS for imaging. As shown in Figure 9A,A’, the bio-fluorescence signals at the 12-week post-injection timepoint were higher at the site of injections (marked in red circles) in the case of shNT in comparison to shOrai3. Moreover, we detected bio-fluorescence signals (identified with yellow arrows) in several parts of mice bodies in the case of shNT Panc1 injections (Figure 9A). However, such signals (identified with yellow arrows) were either absent or considerably weaker in the case of shOrai3 Panc1 stables injected mice (Figure 9A’). This observation suggests that Orai3 silencing substantially decreases PC metastasis in the mice model.

As most of the PC deaths are associated with liver metastasis, and Orai3 expression is inversely correlated with patient survival time (Figure 1), we evaluated the role of Orai3 in driving thoracoabdominal metastasis. For these studies, we covered the site of primary xenografts with black sheet and focused on the thoracoabdominal metastasis. As expected, we observed noticeably higher bio-fluorescence intensity (marked in red circles) in the case of shNT Panc1 stables injected mice (Figure 9B) in comparison to the shOrai3 Panc1 stables injected group (Figure 9B’) suggesting that Orai3 plays a critical role in PC metastasis. We next performed studies for identifying potential mechanisms functioning downstream of Orai3 for regulating PC metastasis. Since metastasis is a very complex phenomenon involving several steps, based on the literature survey suggesting a role for SOCE in regulating MMP expression and EMT [42,43,44,45], we only focused on MMP expression and EMT in this study. Recently, STIM1-Orai1 mediated SOCE was shown to regulate expression of key metastasis associated MMP2 expression [43] in cancerous cells. We thus examined the expression of MMP2 in shNT and shOrai3 tumor lysates. As presented in Figure 9C,D, Western blot analysis of MMP2 protein expression in tumor lysates shows that MMP2 levels are significantly decreased in shOrai3 Panc1 tumors in comparison to shNT Panc1 tumors. We next evaluated levels of E-cadherin, an epithelial marker whose levels are inversely associated with EMT, in shNT and shOrai3 tumor lysates. Western blot analysis of E-cadherin levels in the tumor lysates shows that E-cadherin levels are considerably lower in shNT tumors as compared to shOrai3 tumors, suggesting a higher level of EMT in shNT condition than that in shOrai3 (Figure 9E,F). These data implicate a critical role of Orai3 in regulating MMP2 expression and EMT progression.

Collectively, the in vivo data elegantly demonstrate that Orai3 plays a crucial role in PC progression and tumor growth. Most importantly, this study establishes Orai3 as a critical regulator of PC metastasis, which is associated with poor prognosis in the case of PC patients.

## 4. Discussion

Pancreatic cancer (PC) is one of the most lethal forms of cancer wherein mortality is majorly associated with metastasis to secondary sites. In this study, we have identified a Ca^2+^ influx channel as a novel regulator of PC metastasis. We demonstrate that Orai3 controls PC progression and metastasis in vivo by modulating cell-cycle progression, MMP2 expression and EMT. Further, our extensive analysis of publicly available datasets clearly shows that higher Orai3 levels in PC tumor samples are associated with the lower mean survival time of PC patients. This suggests that Orai3 could be an attractive therapeutic target for the clinical management of PC.

Considering that a large spectrum of cellular functions including cell proliferation and migration are regulated by Ca^2+^ signaling, it is not surprising that it plays a critical role in PC tumorigenesis. One of most critical Ca^2+^ influx pathways in epithelial cells is SOCE, which is mediated by Orai channels. We demonstrate that Orai3 is overexpressed in a subset of PC cell lines (Figure 1) and it encodes a functional Ca^2+^ entry channel in Panc1 cells (Figure 3E–H). An earlier study has reported that Orai1 also contributes to SOCE in Panc1 cells; however, the role of Orai3 in Panc1 cells was not evaluated in that work [46]. Recently, it was suggested that several forms of homo- and hetero-multimeric Orai channel complexes can contribute to functional Ca^2+^ influx pathways in the same cell type [17,47,48]. It was further demonstrated that the expression profile of the channel proteins and agonist strengths control the activation of a particular channel complex over the other [47,48]. Therefore, the contribution of Orai1 to Ca^2+^ entry in PC cells cannot be ruled out and Orai1 could be regulating Ca^2+^ influx under certain specific cellular conditions by constituting hetero-multimeric SOCE channels with Orai3. Indeed, it was recently reported that Orai1 and Orai3 can encode a hetero-multimeric Ca^2+^ entry channel in MiaPaCa2 cells [33]. In this study, authors observed an increase in Ca^2+^ influx upon Orai3 silencing in MiaPaCa2 cells. A possible explanation for the contradictory effect on Ca^2+^ entry could be expression level of Orai3 in these cells. Our data suggest that Orai3 protein expression (Figure 1D,E) as well as SOCE and 2-APB potentiation (Figure 2C,I,J) is relatively low in MiaPaCa2 cells. Therefore, Orai3 may be constituting a hetero-multimeric channel in these cells. Based on this study and our data, it can be hypothesized that Orai3 can form hetero-multimeric SOCE channels in pancreatic cancer cells. However, if Orai3 expression is above a certain threshold level, it can constitute homomeric SOCE channels. Here, we have specifically focused on the role of Orai3 in Panc1 cells since Orai3 is overexpressed in these cells. Our standard SOCE measurement experiments with shRNA mediated stable silencing of Orai3 clearly demonstrates that Orai3 is a critical regulator of Ca^2+^ entry in Panc1 cells (Figure 3E–H). Moreover, 2-APB-mediated potentiation of SOCE (a hallmark signature of functional Orai3 channel) in non-transfected wild-type Panc1 (Figure 2E) and shNT Panc1 stable cells (Figure 3E) corroborates a significant role of Orai3 in mediating Ca^2+^ influx in these cells. Importantly, Orai3 knockdown led to a substantial decrease in the 2APB-mediated potentiation of SOCE in Panc1 (Figure 3F,H) implicating that, indeed, Orai3 contributes to SOCE in these cells. Using concatenated tetrameric Orai1/Orai3 channels, it was recently reported that 2-APB can stimulate SOCE potentiation of heteromeric Orai1–Orai3 SOCE channels [49]. Therefore, the potential contribution of Orai1 to heteromeric SOCE channels in PC needs to be further investigated in future studies.

Our functional assays in Panc1 cells with stable knockdown of Orai3 identified a critical role of Orai3 in cell proliferation and migration (Figure 5 and Figure 7). Our detailed mechanistic studies suggested that Orai3 regulates cell-cycle progression, MMP2 expression and EMT, which in turn contributes to cell proliferation and migration. We observed that Orai3 silencing leads to cell-cycle arrest in the G_1_ phase (Figure 5). Several earlier studies have also reported an important role of Orai3 in G_1_ phase transition in breast, lung and prostate cancer cells [25,32,40]. Interestingly, in breast and lung cancer cells, Orai3 was demonstrated to encode a homomeric SOCE channel whereas in prostate cancer cells Orai3 was shown to constitute a heteromeric ARC channel [25,32,40]. Further, in all these studies Orai3 silencing led to a decrease in the expression of key G_1_ phase transition regulators, i.e., cyclin D1 and Cdk4 [25,32,40]. We also observed that stable knockdown of Orai3 in Panc1 results in significant decrease in the cyclin D1 and Cdk4 levels (Figure 5 and Figure 8; Please refer Figure S3 for full western blot images). Collectively, our data and earlier published studies suggest that independent of the type of Ca^2+^ entry channel encoded, Orai3 plays a vital role in G_1_ phase transition by controlling the expression of cyclin D1 and Cdk4.

Most notably, we carried out extensive in vivo studies in immune-compromised mice for investigating role of Orai3 in PC progression and metastasis. Our in vivo data elegantly demonstrates a critical role of Orai3 in PC tumor growth and metastasis (Figure 8 and Figure 9). Earlier work from our group and others have demonstrated that Orai3 plays an important role in tumor progression in vivo [24,32,33]. We were first to show that Orai3 regulates breast cancer tumorigenesis in vivo [24]. Subsequently, Orai3 was reported to contribute to prostate and more recently to PC tumor growth [32,33]. Here, we evaluated the contribution of Orai3 to pancreatic cancer development and found that Orai3 silencing drastically decreases tumorigenesis in vivo. Interestingly, in earlier studies Orai3 was reported to encode diverse form of Ca^2+^ influx channels in different cancer types, i.e., homomeric SOCE channel in estrogen receptor expressing breast cancer cells [23,24,25]; heteromeric ARC channel in LNCaP prostate cancer cells [32] and very recently heteromeric SOCE channel in MiaPaCa2 cells [33]. Similarly, our data suggests that Orai3 constitutes a SOCE channel in Panc1 cells and thereby regulates Panc1 tumorigenesis. An interesting observation that collectively emerges from the earlier studies and this work is that independent of the kind Ca^2+^ influx channel constituted, Orai3 plays an integral role in tumorigenesis [24,32,33]. An important point is that Orai3 is overexpressed in all these cancers (cancerous cells used in the in vivo studies) and Orai3 silencing in these cells leads to a decrease in their tumorigenic potential. A thought-provoking question emerging out of these studies is “can Orai3 regulate cancer progression (at least partially) independent of its role in Ca^2+^ influx pathways?” Certainly, future studies aimed at understanding detailed molecular mechanisms connecting Orai3 and tumorigenesis are needed to address this intriguing query.

It is important to highlight that although Orai3 is reported to regulate cell migration in a variety of cancerous cells [21], the significance of Orai3 in driving cancer metastasis is not investigated earlier. Moreover, the majority of PC associated deaths are due to metastasis. Therefore, we used GFP-tagged stable Orai3 silenced Panc1 cells for evaluating the role of Orai3 in primary tumor growth and secondary metastatic spread. We observed that shOrai3 Panc1 stables injections in NOD SCID mice lead to a drastic decrease in the tumor growth as compared to that in case of shNT Panc1 stable injections (Figure 8). For examining the relevance of Orai3 in PC metastasis, we traced GFP-positive Panc1 cells using live animal bio-fluorescence imaging. We detected substantially weaker bio-fluorescence signals in the thoracoabdominal region (marked in red circles) in the case of shOrai3 Panc1 stables in comparison to control shNT Panc1 stables (Figure 9B,B’). Further, the overall tumor spread (identified with yellow arrows) was considerably higher in shNT Panc1 stables injections in comparison to shOrai3 Panc1 stables injections (Figure 9A,A’). These data clearly implicate that Orai3 plays a vital role in PC metastasis to the secondary sites.

Collectively, our in vivo studies suggest that Orai3 regulates PC metastasis (Figure 9). Indeed, the role of Orai channels mediated SOCE in driving cancer cell migration and metastasis is starting to emerge in a variety of cancer types [44,45,50]. However, molecular mechanisms driving metastasis downstream of SOCE remain poorly understood. Cancer metastasis is an extremely complex phenomenon that involves several steps, such as cell invasion, EMT, intravasation and extravasation at secondary metastatic sites. Recently, STIM1-Orai1 mediated SOCE was reported to control MMP2 expression and the recycling of MT1-MMP thereby regulating cell invasion [42,43]. Similarly, Orai3 was recently demonstrated to regulate EMT in breast cancer cells by modulating the expression of key EMT transcription factor Snail [51]. Snail promotes EMT by repressing the expression of E-cadherin, a key epithelial marker and regulator of EMT [52]. Moreover, Orai3 expression was reported to be upregulated in the mesenchymal subtype of breast cancer cells suggesting that higher Orai3 levels are associated with the mesenchymal nature of cancerous cells [53]. Based on this literature, we evaluated Orai3′s role in regulating the expression of MMP2 and E-cadherin during PC metastasis. Our data clearly demonstrates that Orai3 silencing results in elevated E-cadherin levels and a concomitant decrease in MMP2 expression in the tumors in vivo (Figure 9). This suggests that Orai3 regulates PC metastasis at least partly by modulating MMP expression and EMT induction in PC cells. Since Orai3 also regulates Panc1 proliferation, the potential contribution of decreased proliferation to the reduction in secondary metastasis cannot be completely ruled out. In any case, we have presented several independent data sets to support our hypothesis that Orai3 can regulate metastasis. First of all, our in vitro migration assays with two autonomous techniques show that Orai3 regulates migration of PC cells. Secondly, our in vivo analysis of MMP2, a critical regulator of cancer cell invasion and metastasis, shows that Orai3 knockdown decreases the expression of MMP2. Further, examination of E-cadherin upon Orai3 silencing suggests that Orai3 modulates EMT, an essential step in cancer metastasis. Therefore, taken together, our data clearly suggest that Orai3 regulates the integral components of cancer metastasis such as cell migration, invasion and EMT. Definitely, further studies are required for comprehensively delineating the molecular choreography that connects Orai3 to cancer metastasis in general and PC progression in particular.

Taken together, we report that Orai3 constitutes a functional Ca^2+^ entry channel in PC cells and it drives PC tumorigenesis in vivo. Most importantly, we demonstrate that Orai3 regulates PC metastasis, which is the primary cause of PC associated deaths. Considering that Orai3 expression is inversely associated with the PC patients’ survival time and it is overexpressed in a certain proportion of pancreatic tumors, Orai3 appears to be an attractive therapeutic target, at least in the cases where its expression is elevated. Certainly, future studies aimed at understanding precise role of Orai3 in PC metastasis and survival are required for devising strategies to target Orai3.

## 5. Conclusions

Orai3 is overexpressed in a large proportion of pancreatic tumors and pancreatic cancer cell lines. Extensive analysis of publicly available datasets suggests that Orai3 overexpression is associated with poor prognosis in PC patients. Our data demonstrate that Orai3 silencing in PC cells decreases cell proliferation and cell migration thereby inhibiting tumor growth and secondary metastasis in vivo. Mechanistically, Orai3 regulates the expression of key G_1_ cell cycle phase regulators, i.e., cyclin D1 and Cdk4. Further, Orai3 modulates expression of MMP2 and E-cadherin for inducing metastatic phenotypes.

## Figures and Tables

**Table 1:** Details of antibodies used in the study.

Protein	Brand Name	Catalog Number
Orai1	Abcam (Waltham, MA, USA)	ab86748
Orai2	Abcam (Waltham, MA, USA)	ab180146
Orai3	Abcam (Waltham, MA, USA)	ab254260
Cyclin D1	Cell Signaling Technology (Denvers, CO, USA)	2978S
Cdk 4	Santa Cruz Biotechnology (Dallas, TX, USA)	sc-601
E-Cadherin	Cell Signaling Technology (Denvers, TX, USA)	24E10
MMP2	Cell Signaling Technology (Denvers, TX, USA)	4022S

**Table 2:** Sequences of primers used in the study.

Gene	Forward Primer	Reverse Primer
*Orai1*	AGGTGATGAGCCTCAACGAG	CTGATCATGAGCGCAAACAG
*Orai2*	GCAGCTACCTGGAACTGGTC	CGGGTACTGGTACTGCGTCT
*Orai3*	TCCCCATCAGTCTGTCCCTT	GAAGGTCCCACAAGCTCTCC
*GAPDH*	AACTGCTTAGCACCCCTGGC	ATGACCTTGCCCACAGCCTT

## Data Availability

Data supporting reported results are available on request from the corresponding author.

## Supplementary Materials

The following are available online at https://www.mdpi.com/article/10.3390/cancers13235937/s1, Figure S1: shOrai3 specifically targets Orai3 without changing Orai1 and Orai2 levels, Figure S2: 2APB stimulated store-independent Ca^2+^ influx is decreased in shOrai3 Panc1 cells, Figure S3: Full Western blot images.
