# Supplementary material for: Orai3 Regulates Pancreatic Cancer Metastasis by Encoding a Functional Store Operated Calcium Entry Channel
Source: Cancers (Basel). 2021 Nov 25;13(23):5937. doi: 10.3390/cancers13235937 (PMC8656723; doi:10.3390/cancers13235937)
Supplement: Supplementary file 1 [file cancers-13-05937-s001.zip › supplementary Figure S1-S2.pdf]

cancers

MDPI

Supplementary Materials: Orai3 Regulates Pancreatic Cancer Metastasis by Encoding a Functional Store Operated Calcium Entry Channel

Samriddhi Arora, Jyoti Tanwar, Nutan Sharma, Suman Saurav and Rajender K. Motiani

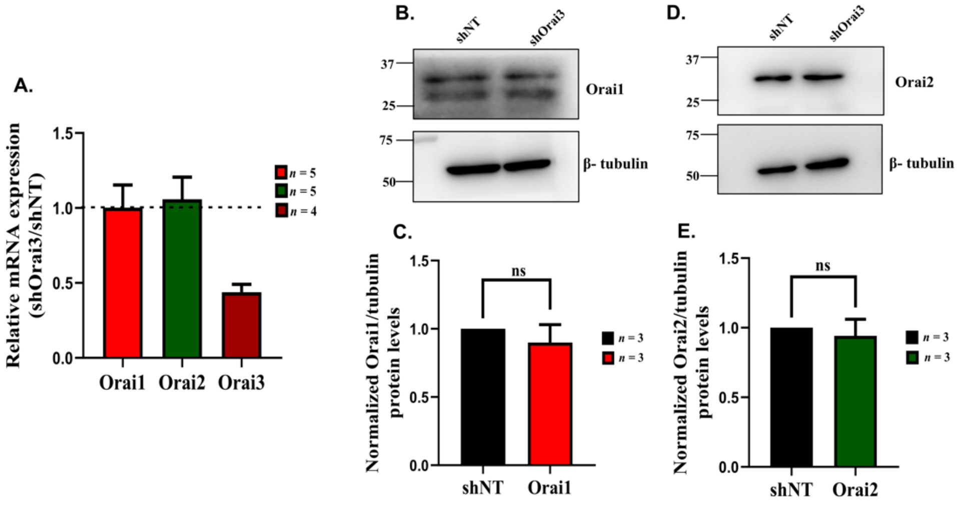

Figure S1. shOrai3 specifically targets Orai3 without changing Orai1 and Orai2 levels. (**A**) qRT-PCRs were performed with the samples arising from shNT and shOrai3 Panc1 stable cells. Orai1, Orai2 and Orai3 mRNA expression (relative to GAPDH) was evaluated using specific primers. shOrai3 decreased Orai3 mRNA levels by over 50% while levels of Orai1 and Orai2 remained unaffected. (**B**) Representative Western blot showing no significant difference in Orai1 protein expression between shNT and shOrai3 Panc1 stable cells. (**C**) Densitometric analysis of Orai1 protein levels in three independent shNT and shOrai3 Panc1 stable clones showing no significant change in Orai1 protein levels. (**D**) Representative Western blot showing no change in Orai2 protein expression between shNT and shOrai3 Panc1 stable cells. (**E**) Densitometric analysis of Orai2 protein levels in three independent shNT and shOrai3 Panc1 stable clones showing no significant change in Orai2 protein levels. Data presented are mean ± S.E.M. Unpaired Student's *t*-test was performed for statistical analysis. *p*-value < 0.05 was considered as significant.

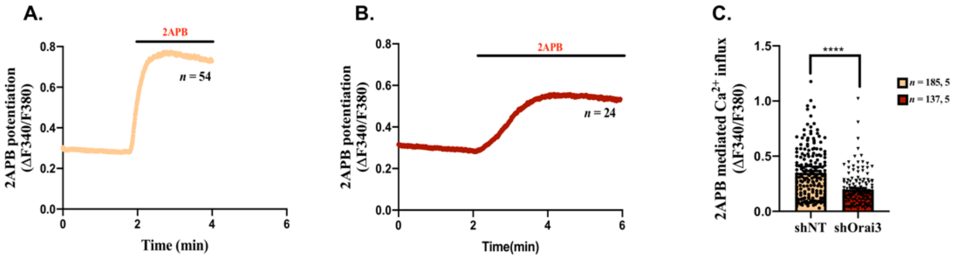

Figure S2. 2APB stimulated store-independent Ca^{2+} influx is decreased in shOrai3 Panc1 cells. (**A**) Representative Ca^{2+} imaging trace of shNT Panc1 stables stimulated with 2APB without pre-exposure of Tg. Here, "$n = 54$" denotes the number of cells in this particular trace. (**B**) Representative Ca^{2+} imaging trace of shOrai3 Panc1 stables stimulated with 2APB without pre-exposure of Tg. Here, "$n = 24$" denotes the number of cells in this particular trace. These experiments were performed in the presence of 2mM Ca^{2+} in the bath solution. (**C**) The extent of 2APB-induced Ca^{2+} influx was

calculated from 185 shNT and 137 shOrai3 Panc1 cells imaged during 5 independent experiments/condition and data are presented in dot plot graphs. Data presented are mean ± S.E.M. Unpaired Student's *t*-test was performed for statistical analysis. p-value < 0.05 was considered as significant and p-value < 0.0001 is presented as "****".

Copyright: © 2021 by the authors. Licensee MDPI, Basel, Switzerland. This article is an open access article distributed under the terms and conditions of the Creative Commons Attribution (CC BY) license (https://creativecommons.org/licenses/by/4.0/).
