# Supplementary material for: Orai3 Regulates Pancreatic Cancer Metastasis by Encoding a Functional Store Operated Calcium Entry Channel
Source: Cancers (Basel). 2021 Nov 25;13(23):5937. doi: 10.3390/cancers13235937 (PMC8656723; doi:10.3390/cancers13235937)

Figure 1D. Orai3 expression in pancreatic cells

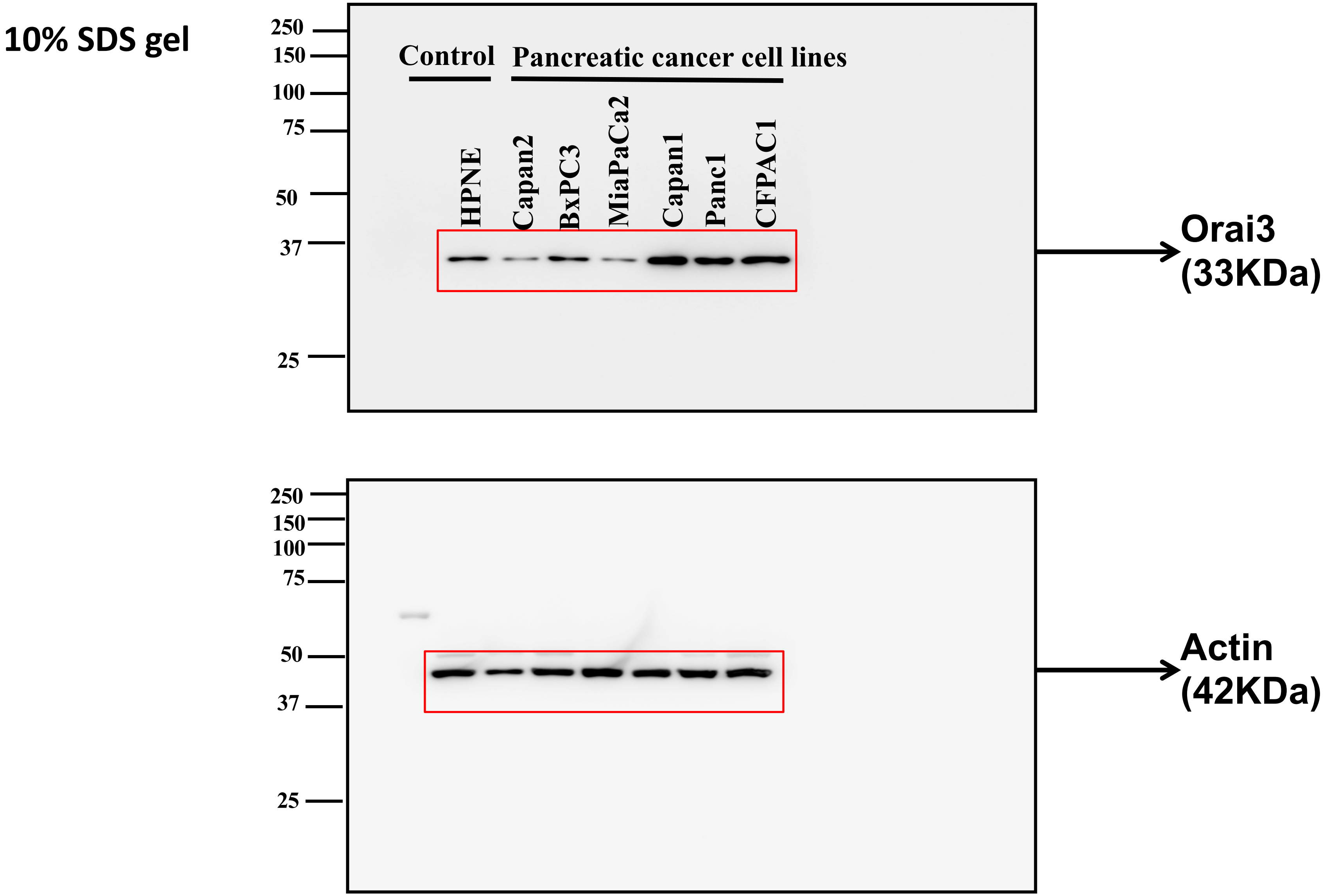

Figure 3C. Panc1 Orai3 knockdown full blots

10% SDS gel

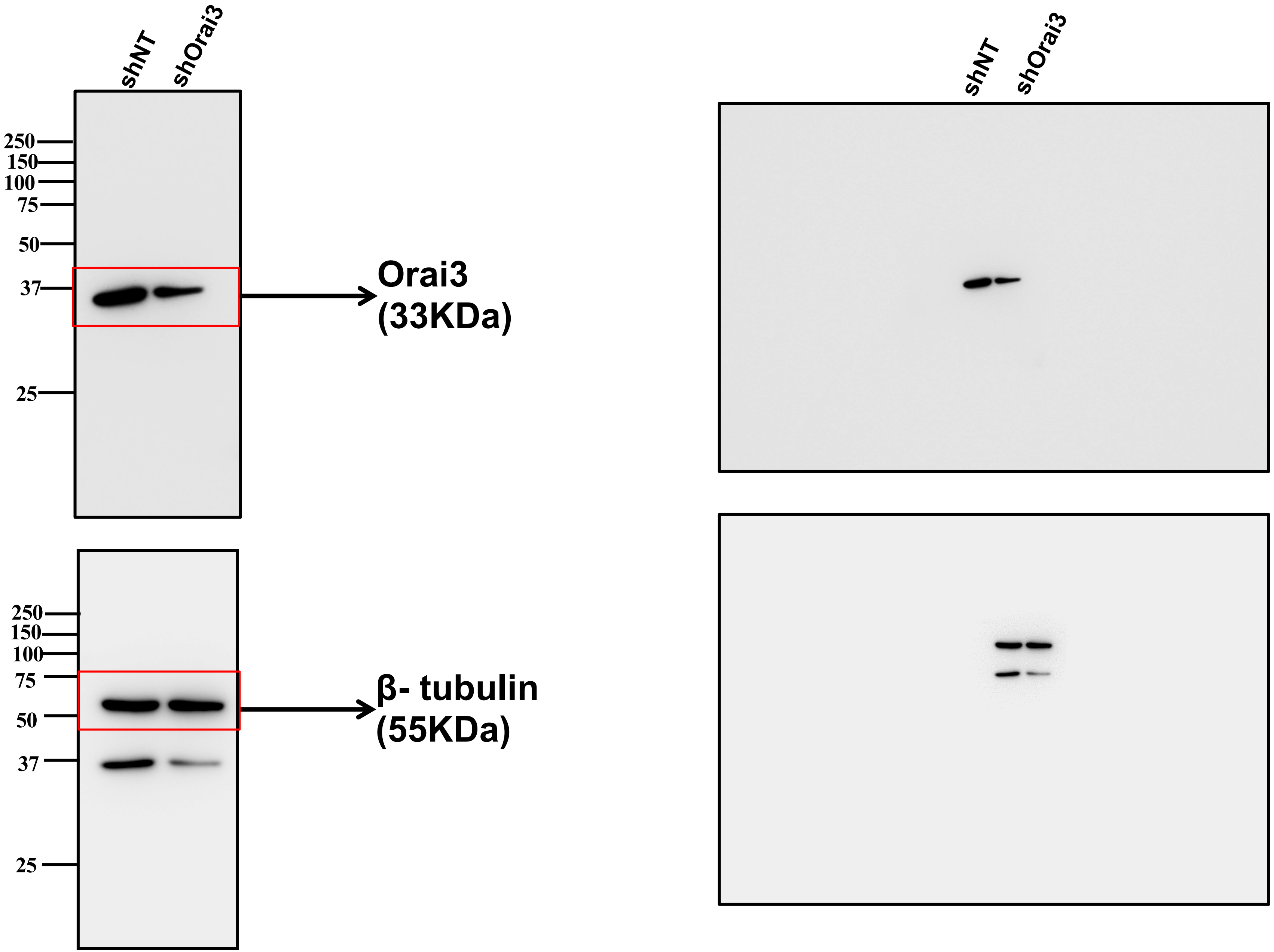

Figure 4D. HPNE Orai3 knockdown full blots

10% SDS gel

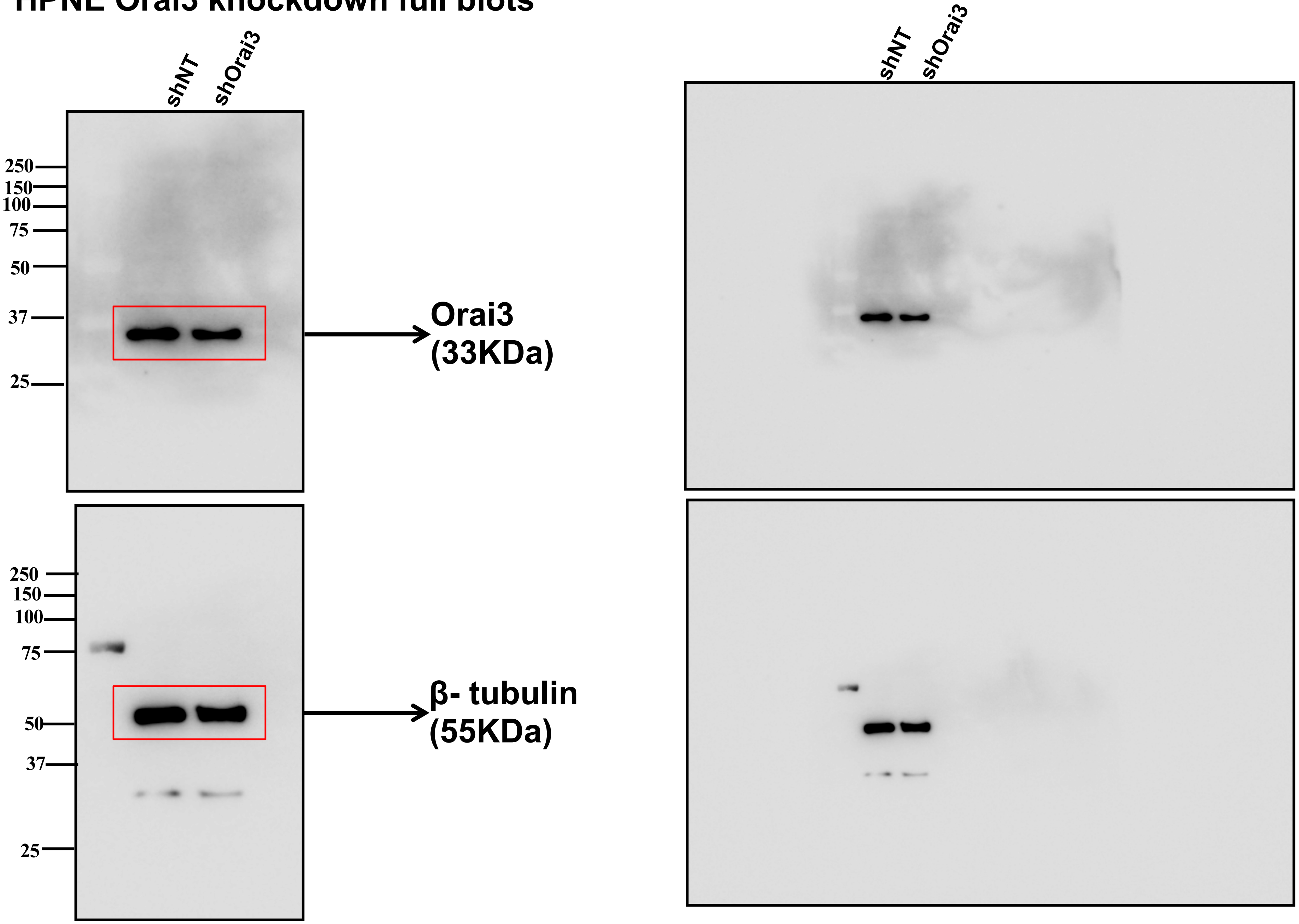

Figure 5D. Cyclin D1

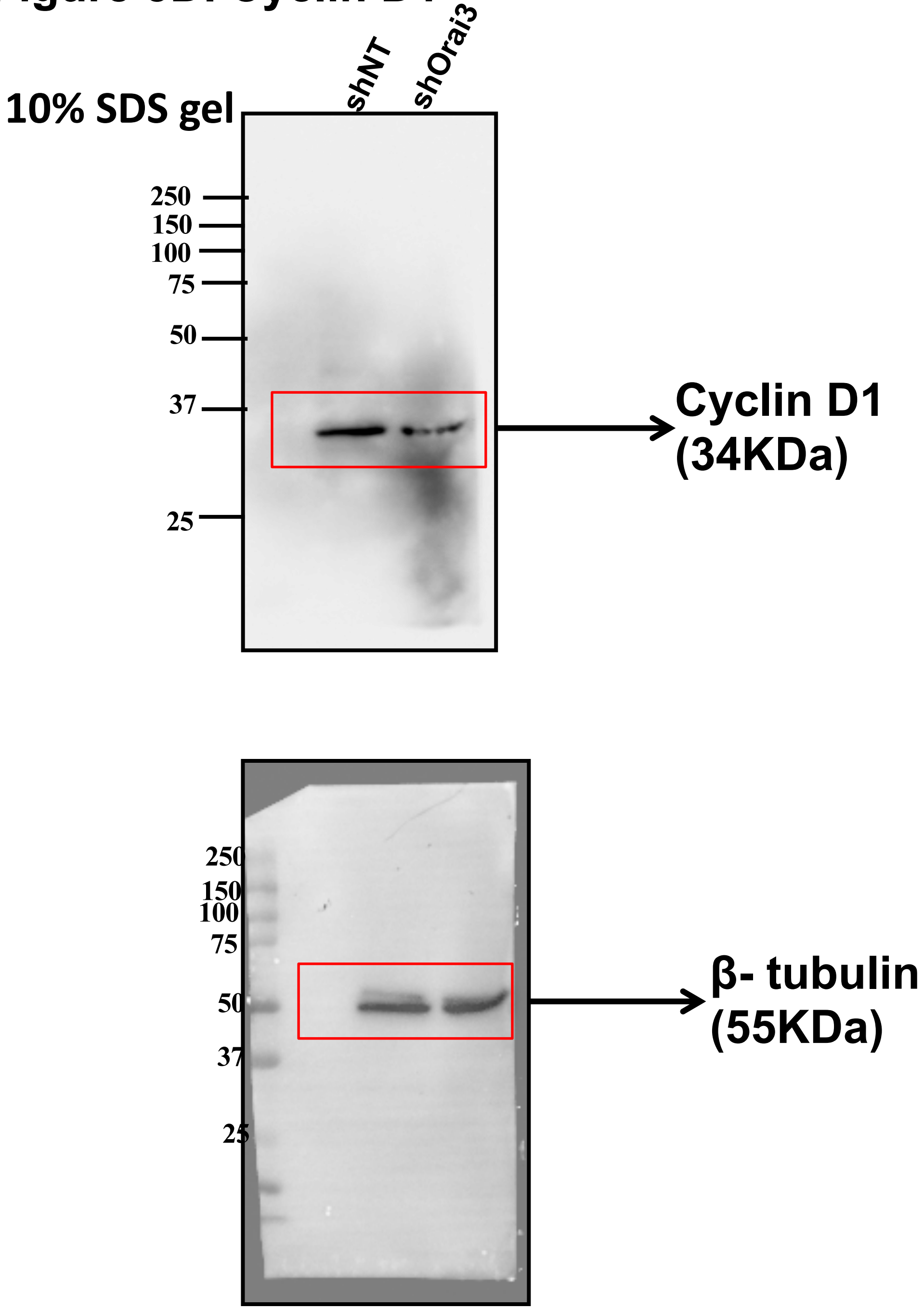

Figure 5F. Cdk4

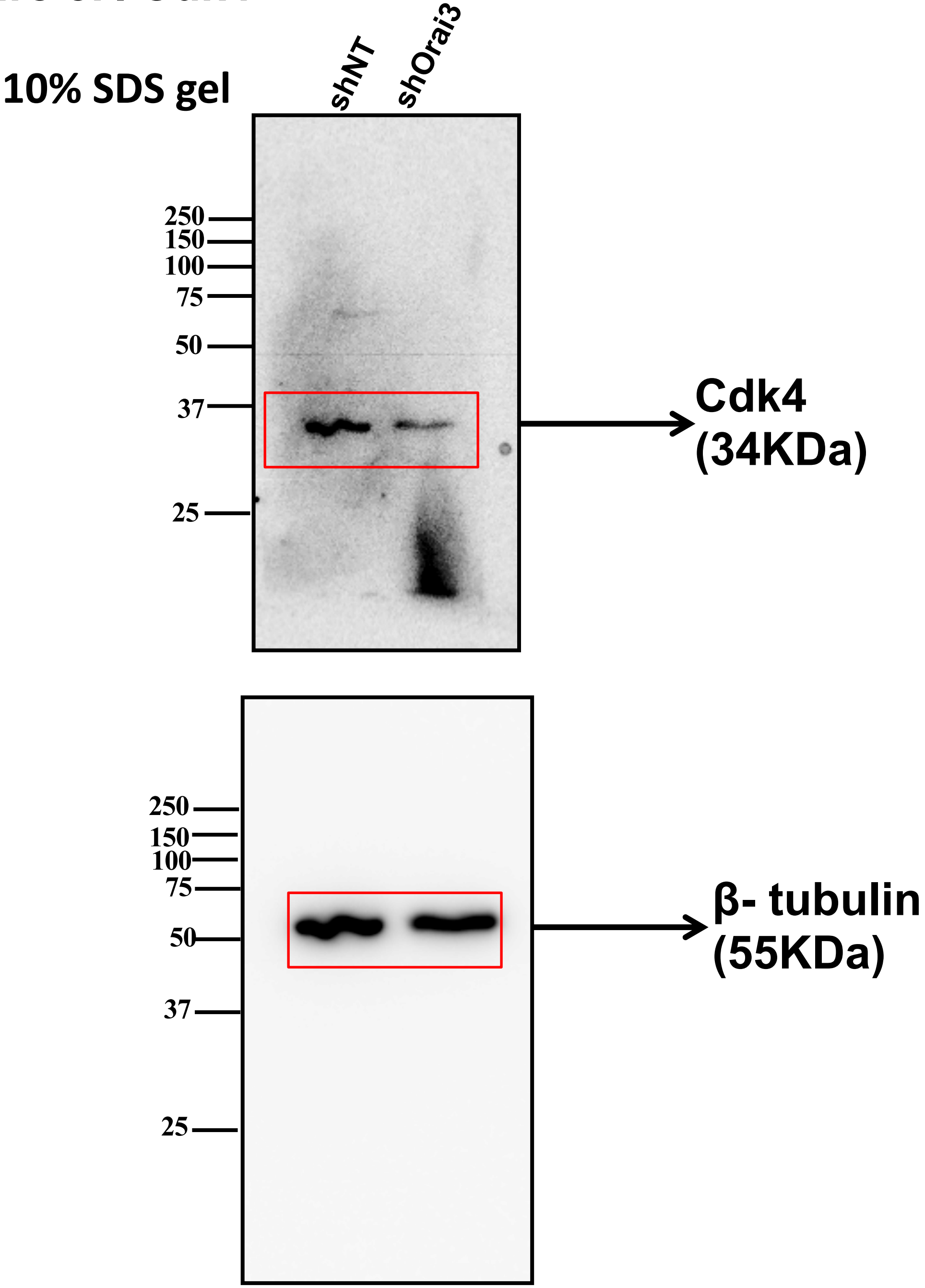

Figure 8D. Orai3 knockdown in tumor samples

12% SDS gel

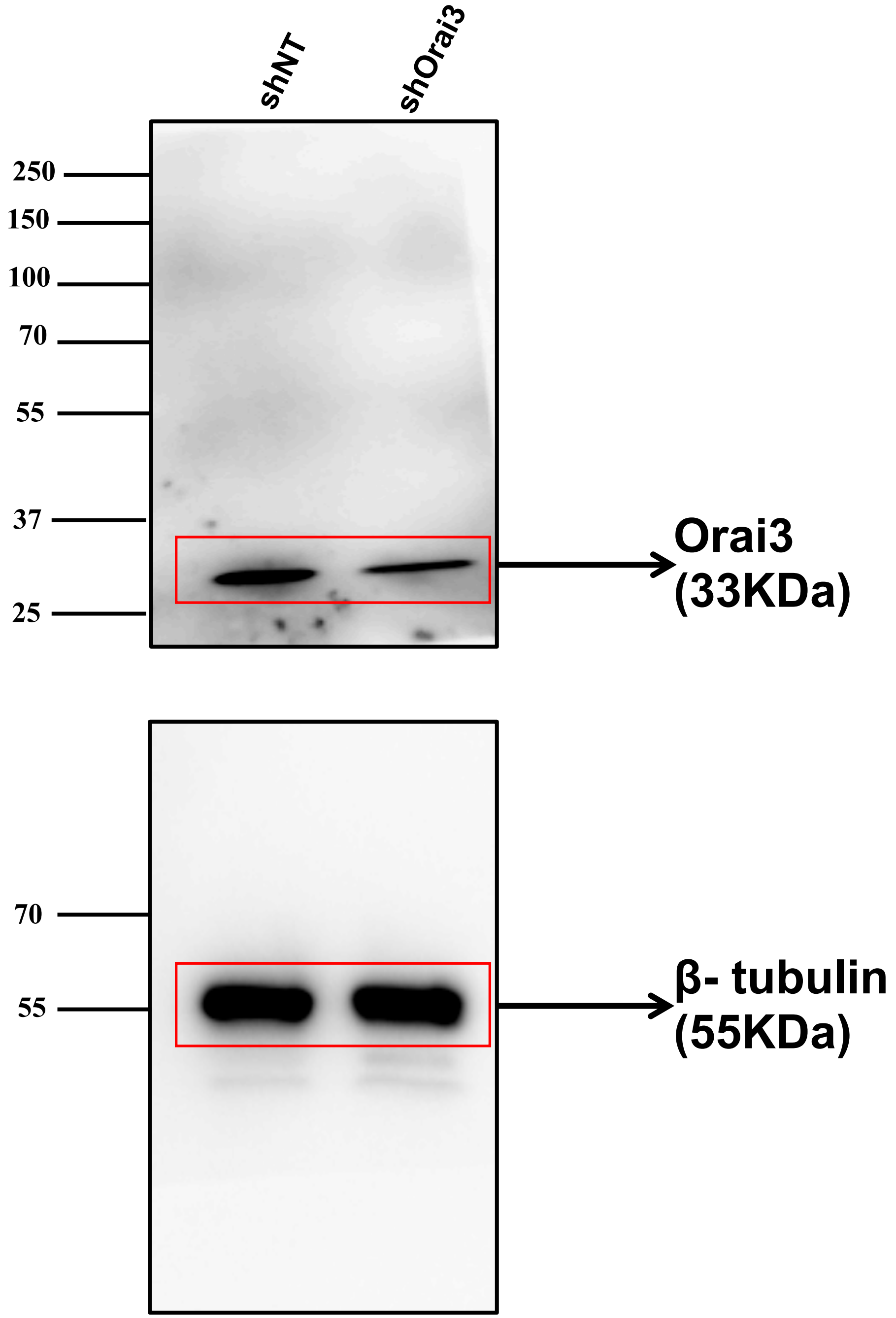

Figure 8F. Cyclin D1 in tumor samples

12% SDS gel

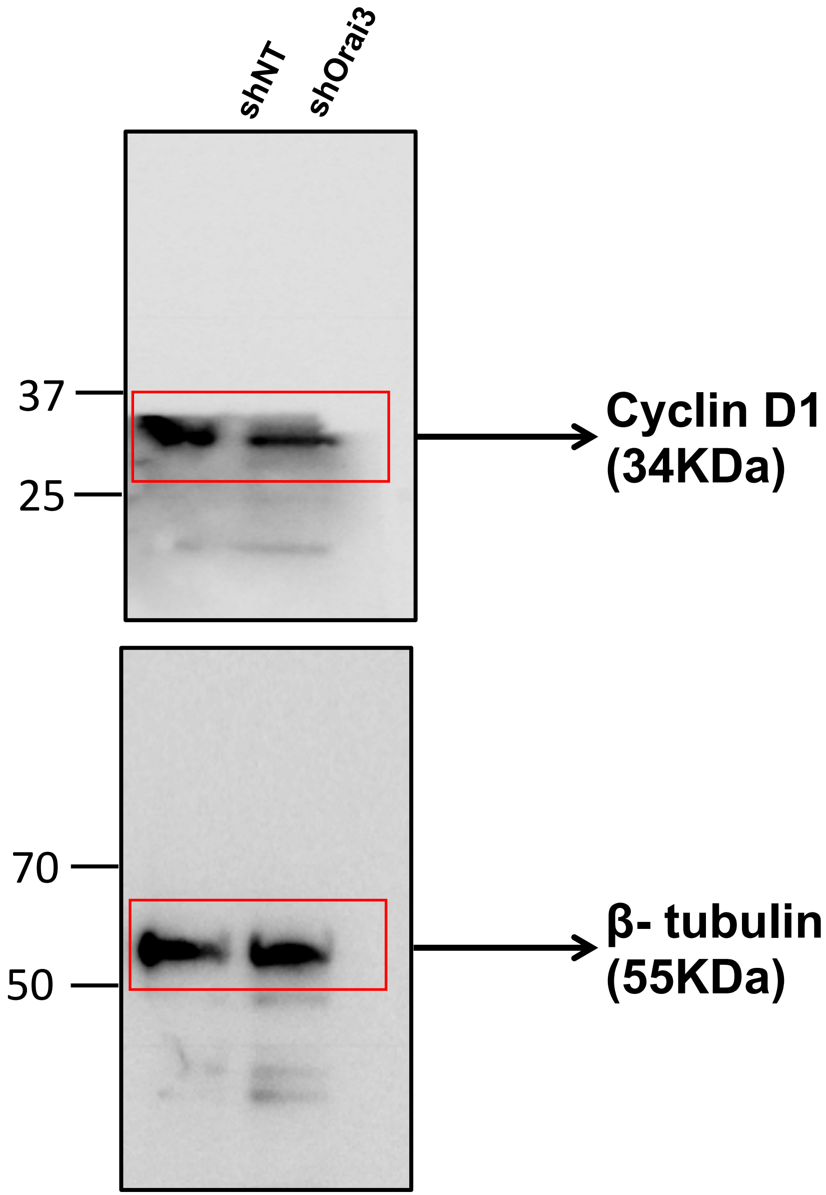

Figure 8H. Cdk4 in tumor samples

12% SDS gel

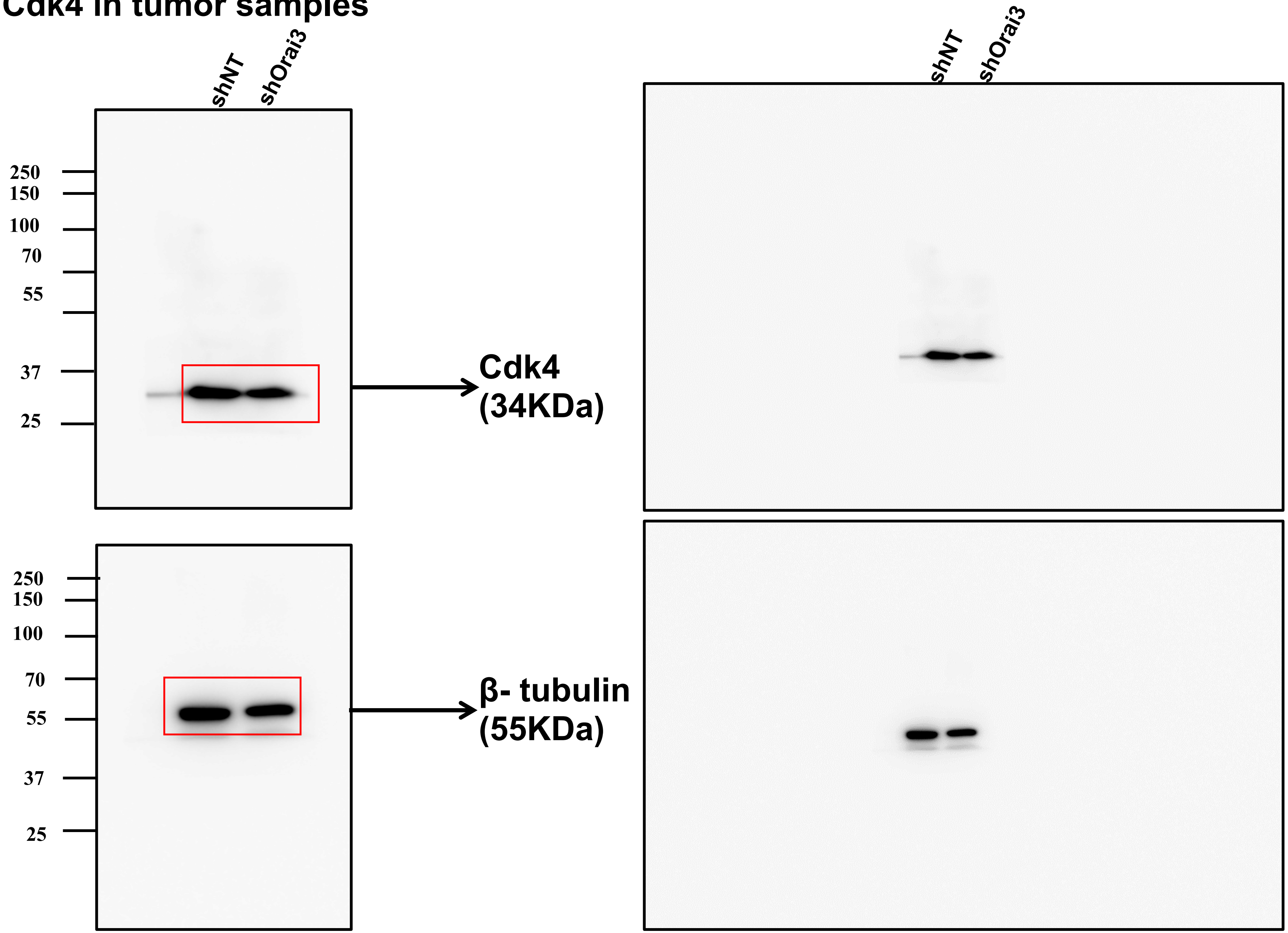

Figure 9C. MMP2 in tumor samples

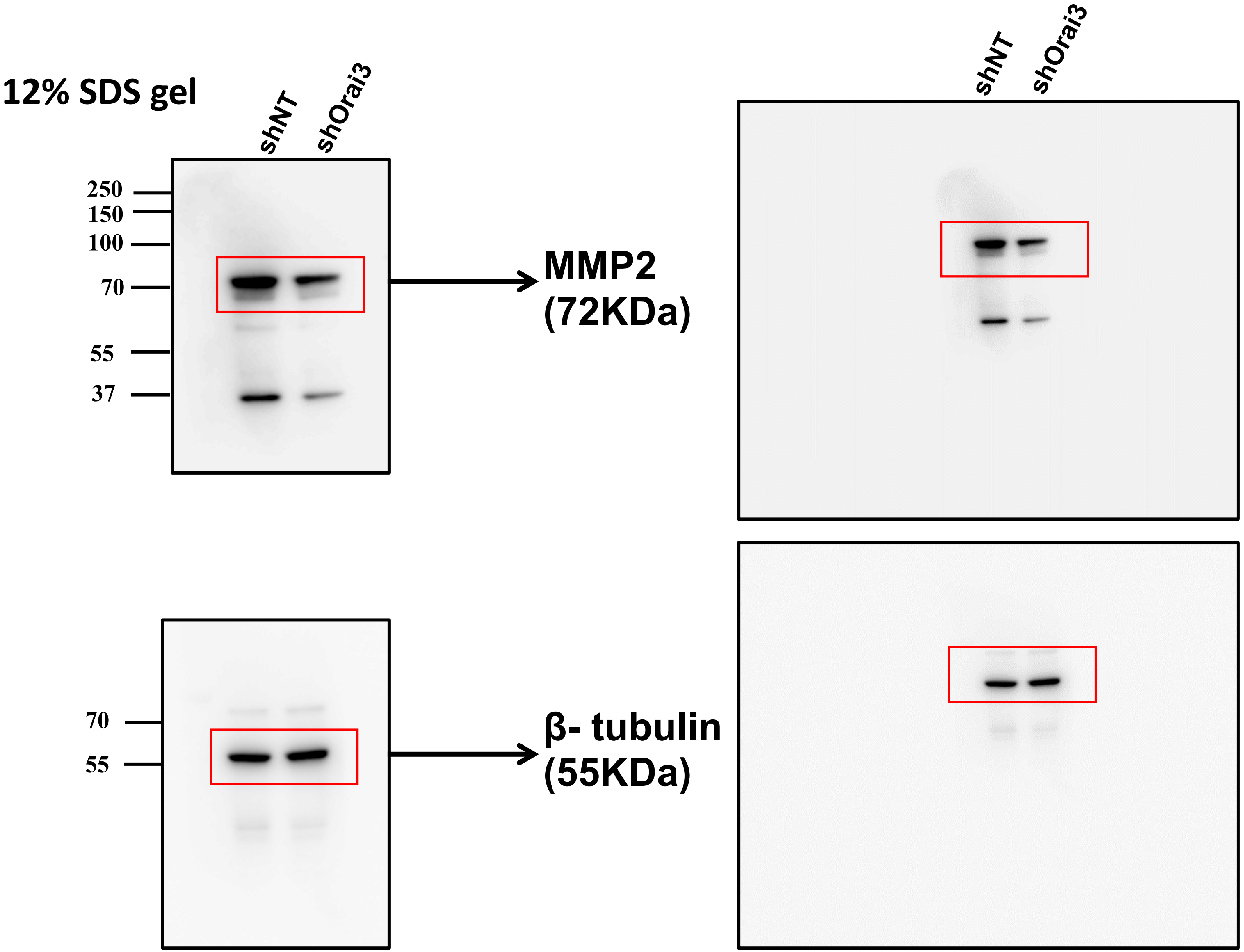

Figure 9E. E-cadherin in tumor samples

8% SDS gel

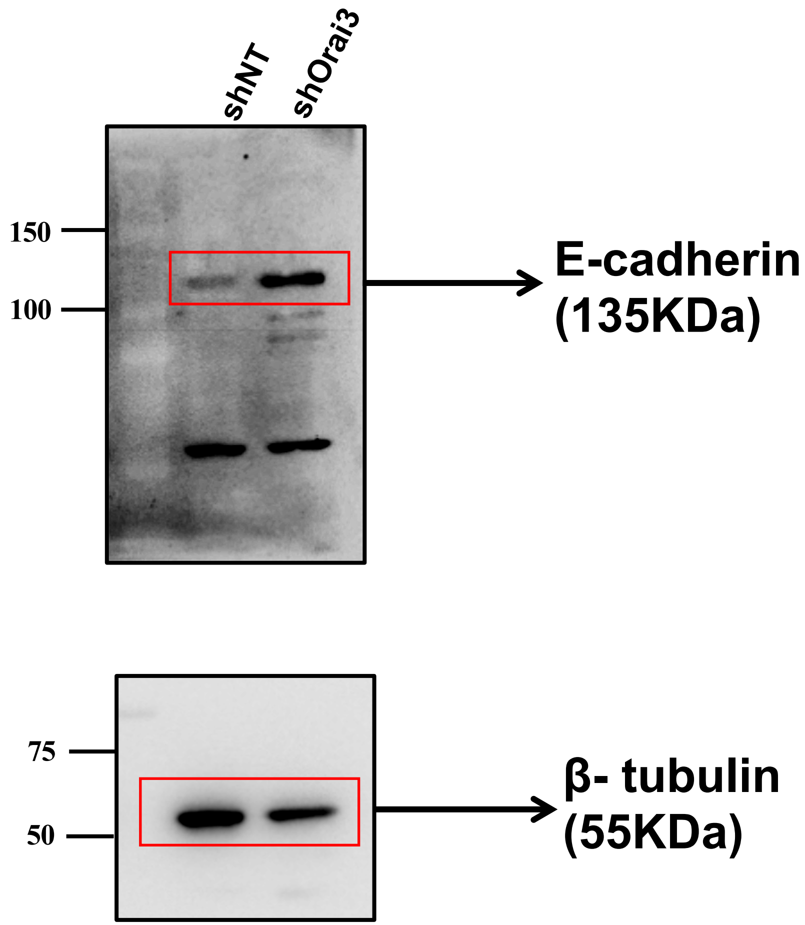

Supplementary Figure 1B and 1C. shOrai3 specifically targets Orai3 without changing Orai1 and Orai2 levels

12% SDS gel

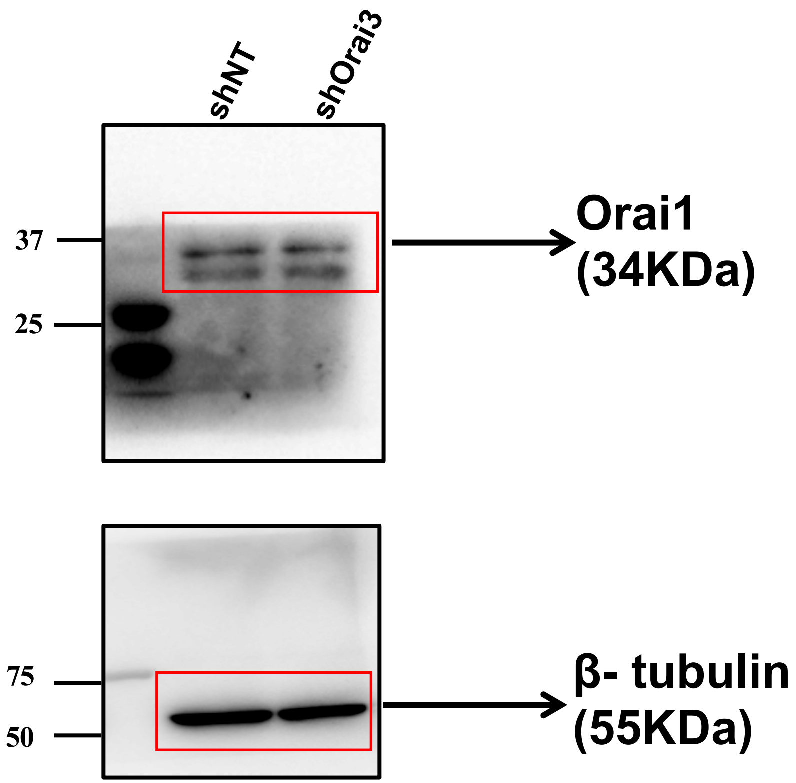

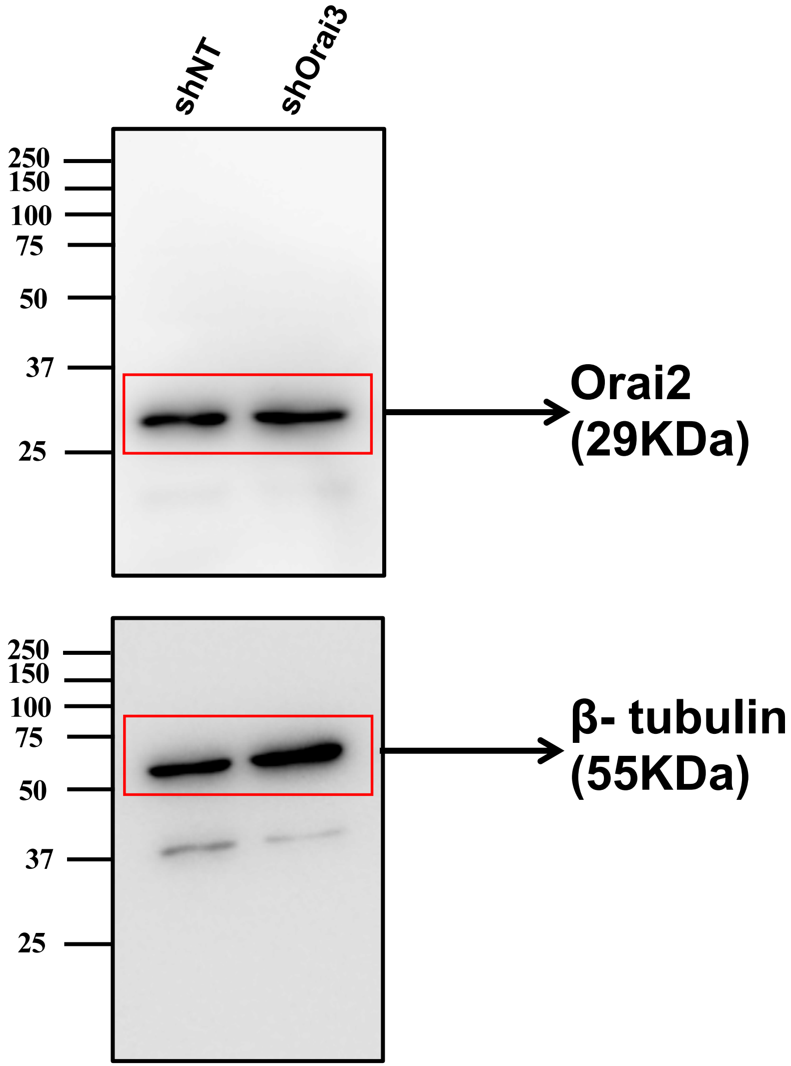

Supplement: Supplementary file 1 [file cancers-13-05937-s001.zip › supplementary Figure S3.pdf]
